# Challenges of safety culture in Surgical Center: mixed methods study ^*^

**DOI:** 10.1590/1518-8345.7001.4206

**Published:** 2024-07-29

**Authors:** Nery José de Oliveira Junior, Caren de Oliveira Riboldi, Daniela Campos de Andrade Lourenção, Vanessa de Brito Poveda, João Lucas Campos de Oliveira, Ana Maria Müller de Magalhães

**Affiliations:** 1 Universidade Federal do Rio Grande do Sul, Escola de Enfermagem, Porto Alegre, RS, Brazil.; 2 Universidade de São Paulo, Escola de Enfermagem, São Paulo, SP, Brazil.

**Keywords:** Patient Safety, Organizational Culture, Perioperative Nursing, Surgicenters, Nursing, Health Facility Environment

## Abstract

**Objective:**

to analyze the safety attitudes of health and support areas professionals working in Surgical Center.

**Method:**

sequential explanatory mixed methods study. The quantitative stage covered 172 health and support professionals in eight Surgical Centers of a hospital complex. The Safety Attitudes Questionnaire/Surgical Center was applied. In the subsequent qualitative stage, 16 professionals participated in the Focus Group. Photographic methods were used from the perspective of ecological and restorative thinking, and data analysis occurred in an integrated manner, through connection.

**Results:**

the general score, by group of Surgical Centers, based on the domains of the Safety Attitudes Questionnaire/Surgical Center, reveals a favorable perception of the safety climate, with emphasis on the domains Stress Perception, Communication in the Surgical Environment, Safety Climate and Perception of Professional Performance. The overall analysis of the domain Communication and Collaboration between Teams appears positive and is corroborated by data from the qualitative stage, which highlights the importance of interaction and communication between healthcare teams as fundamental for daily work.

**Conclusion:**

the perception of safety attitudes among health and support professionals was positive. The perception of the nursing team stands out as closer or more favorable to attitudes consistent with the safety culture.

## Highlights

**(1)** Communication and teamwork reinforce the safety culture.

**(2)** The nursing team is more committed to the safety culture.

**(3)** Favorable perception of the safety climate by health professionals and support workers.

**(4)** Adherence of the nursing team to institutional processes and routines.

## Introduction

 The World Health Organization highlights that every year, in low- and middle-income countries, approximately 134 million adverse events and 2.6 million deaths associated with unsafe environments occur, proving that patient safety must be a permanent goal. In this context, although there are advances in evidence on the impact of adverse events, measures to reduce, mitigate, prevent and measure potential risks in terms of technology, financial resources and interventions are still incipient ( ^1^ ). For improvements to be implemented, it is necessary to foster a culture that favors patient safety in healthcare organizations ( ^2^). 

 Safety culture is understood as a set of values and individual and collective practices focused on reducing risks and harm to patients ( ^3^ ). As it is a cultural and eminently multifaceted element, which emanates from an organizational conception, there are factors considered measurable ^(^ ^4^ ^)^ . One example is the safety climate, referring to the perception of organizational actors about (un)safe policies and practices in the healthcare environment ^(^ ^3^ ^-^ ^4^ ^)^ . 

 The means of analyzing culture and safety climate include questionnaires, interviews and observations, or even external assessment by subject matter experts. Such analyzes allow verifying the level of institutional and professional commitment to values, beliefs, resources, attitudes and behavior related to patient safety ^(^ ^1^ ^,^ ^4^ ^-^ ^5^ ^)^ . In other words, even if in practice safety climate is assessed through work dynamics, including mainly the perception of workers, this will be a reflection of how the organizational culture positions itself regarding safe care ^(^ ^2^ ^,^ ^5^ ^)^ . 

 Research related to safety culture in the hospital environment is constantly growing ^(^ ^6^ ^-^ ^7^ ^)^ , which reinforces the fact that organizational values directly impact safety results, also encompassing the occurrence of adverse events, that is, concrete harm to patient ^(^ ^8^ ^)^ . Such values need to be strengthened in any care delivery environment, even though it is recognized that some environments have a higher or more evident risk due to the nature of the work processes developed, such as the Surgical Center (SC) ^(^ ^9^ ^)^ . 

 So-called *near misses* or *almost errors* were less frequent in SC than in other units, such as hospitalization, intensive care and pediatrics ^(^ ^10^ ^)^ . On the other hand, the occurrence of adverse events of very high severity — known as *never events* — is alarming when it comes to the intraoperative and immediate post-operative period, such as surgery in the wrong laterality, wrong procedure and/or patient, retention of material inside the patient and electrocautery burns ^(^ ^11^ ^)^ . Added to this is the fact that up to 90% of adverse events related to surgery are classified as preventable ^(^ ^12^ ^)^ , making it imperative that patient safety measures in the SC are instituted and encouraged, which can be accomplished with greater assertiveness through systematic analyzes of safety attitudes in these environments. 

In view of the above, this research was based on the following guiding questions: What is the perception of the safety climate among health and support areas professionals working in SC? What aspects have an impact on the safety culture in this scenario? Therefore, the objective of this work was to analyze safety attitudes among health and support areas professionals working in SC.

## Method

### Study design

 This is a mixed methods study, anchored in the sequential explanatory design, which combines quantitative and qualitative elements to answer the research questions in a more comprehensive and complete way. The first phase, with a quantitative approach and greater weight (QUAN), allowed specific variables to be measured objectively, while the subsequent stage, with a secondary qualitative approach (Qual), aimed to deepen the understanding of the previously measured phenomenon. The initial analysis of the quantitative results guided the collection of qualitative data (QUAN→Qual), supporting insights to be explored in greater detail and facilitating the discovery of new meanings, interpretations and relations between variables ^(^ ^13^ ^)^ . 

### Data collection scenario

The study was conducted in a single complex composed of eight hospitals, located in the city of Porto Alegre-RS, Brazil. Each location has a SC, totaling 53 rooms dedicated to general and specialty surgeries. In total, surgical productivity is approximately 6,000 procedures per month. The SC included in this research are characterized as follows: SC “A” (13 rooms), which performs surgeries of different specialties; SC “B” (4 rooms), intended for ophthalmological procedures; SC “C” (3 rooms), which performs pulmonology specialty surgeries; SC “D” (3 rooms), which performs neurological surgeries; SC “E” (4 rooms), which performs cardiac surgeries; SC “F” (7 rooms), which serves the oncology specialty; SC “G” (12 rooms), which covers the specialties of plastic surgery and transplants; and SC “H” (7 rooms), which serves the specialty of pediatrics.

### Period

The study took place between June 2020 and February 2021.

### Population

The study population consisted of health and support areas professionals from the eight SC. The health professionals were: surgeons and anesthesiologists, medical residents, nurses and nursing technicians. Administrative assistants, pharmacy and hygiene assistants were considered as support areas.

### Selection criteria

The eligibility criteria for participation in the study consisted of being part of the team of one of the SC and not being on vacation or functional leave during data collection.

### Participants

The quantitative stage covered 172 professionals, distributed among the following categories: surgical scrub and circulating nurse (100), SC nurse (22), surgeon or assistant surgeon (16), surgery resident or intern (9), head nurse (7), administrative assistant (6), anesthesiologist (5), perfusionist (3), anesthesiology resident (1) and pharmacy or hygiene assistants (3). It should be noted that there was a low number of surgeries being performed due to the restrictions during the Covid-19 pandemic period, which resulted in a lower presence of medical teams, as surgical schedules were reduced. These restrictions also impacted the subsequent qualitative stage, which had 16 participants including: nurses (5), nursing technicians (10) and administrative assistant (1). For this phase, in addition to participating in the previous stage, it was necessary to work on the SC that presented the most favorable or unfavorable scores in the quantitative stage. In the first meeting, eight representatives from each SC participated, and in the second, 12 workers, six from each group.

### Instruments used to collect information

In the quantitative stage, the Safety Attitudes Questionnaire/Surgical Center (SAQ/SC) was used, with the purpose of measuring the safety climate in health services based on the professionals’ perception of patient safety. The researchers opted for a less updated version of the instrument, because culturally, in the place where the study was carried out, nursing technicians perform the role of circulating or scrub alternately on the work schedule. In the current version of the instrument, these functions were grouped together, not applying to the reality in question.

 The Brazilian version of the SAQ/SC consists of a Likert-type scale and is divided into three parts. The first, with 15 statements, refers to the quality of communication and collaboration between professionals who work in the surgical environment, which the research participant must answer about their relationship with each of the professional categories. The second is composed of 40 statements, conceptually divided into six domains: safety climate (seven items), management perception (five items), stress perception (four items), working condition (six items), communication in the surgical environment (four items) and perception of professional performance (four items). The third part covers demographic information (gender, race/ethnicity, professional category, length of experience, work shift, among others) and a space in which the participant can write three recommendations for improving patient safety in SC, and indicate if he had already answered the instrument previously ^(^ ^14^ ^)^ . 

 The subsequent qualitative stage used the Focus Group (FG) technique and photographic research methods from the perspective of ecological and restorative thinking, through photographic walks (PW) ^(^ ^15^ ^-^ ^16^ ^)^ . At this stage, the sample was intentional and for convenience, among those who participated in the first stage (quantitative). 

To conduct the FG, a script prepared by the researchers was used, covering the topics: safety culture, what influences the safety culture, the relationship of the multidisciplinary team in the SC environment, and the safety climate in the SC.

The FG technique was organized and conducted according to the following script: opening of the session, integration of participants, explanation of the dynamics of discussions, group setting, debate, synthesis of previous moments and closing of the meeting. Furthermore, an agreement on confidentiality was signed, reinforcing that the debates and ideas discussed in the meetings would be restricted to group members.

The script was planned based on the need to complement/deepen the quantitative findings. Therefore, the investigation of these elements was important to constitute a qualitative database, creating a repository of rich and contextual information that can be verified, to find inferences and clarifications about the quantitative findings.

### Data collection

In the first phase of the study, collecting quantitative data, the questionnaire was administered in printed form and in person, during the professionals’ workday. Convenience sample selection was followed, from June to July 2020. The second phase of the research, of a qualitative nature, took place between October 2020 and February 2021. The sample in this stage was intentional and for convenience, selected among those who participated in the first stage (quantitative).

A list was made available for those who were interested in participating in the FG and in the PW in the SC, defined according to the best and worst results in the SAQ/SC questionnaire. The first 12 registrants for each FG were selected, and four meetings were held, two with SC professionals with the best score (group A) and two with those linked to the location with the worst score (group B).

The first FG meeting was to discuss topics of interest, related to aspects of safety culture and to survey topics considered priorities by the participants, in order to compose the PW roadmap. After the first FG, the topics covered were validated by a member of each group (nurse), who subsequently followed the PW through the SC. The second meeting had as its main focus the discussion of the photos obtained, characterized as FG for photo-elicitation.

### Data processing and analysis

For analysis purposes, the SC were arranged into five groups (A+B; C+D+E; F; G; H), according to the total number of respondents, similarity of processes, number of operating rooms and volume of procedures. The quantitative data from the first stage were analyzed using the Statistical Package for the Social Sciences version 21.0 software. Descriptive statistics were used to characterize the sample, through which discrete variables were presented as mean and standard deviation or median and percentile, and categorical variables were expressed as absolute and relative frequencies.

 For the statistical analysis, the scores of the SAQ/SC domains were considered as dependent variables: Safety Climate, Management Perception, Stress Perception, Working Condition, Communication in the Surgical Environment and Perception of Professional Performance; and as independent variables: age, gender, profession and time of experience in the specialty. The Chi-Square and Shapiro-Wilk tests were used to establish associations between groups and verify sample normality, respectively. The significance level adopted for statistical tests was 5% (p ≤ 0.05), based on the Kruskal-Wallis, Fisher’s exact and Dunn-Bonferroni *post hoc* tests. The reliability of the instrument was measured through internal consistency with the calculation of Cronbach’s alpha coefficient, reaching a satisfactory value of 0.86. 

 In the SAQ/SC results for the quality of communication and collaboration between professionals, simple statistics were adopted. It is important to mention that scores relating to safety climate can range from zero (worst perception of safety climate) to 100 (best perception of safety climate), with values greater than or equal to 75 being considered a positive perception of patient safety ^(^ ^17^ ^)^ . The lower the score, the more fragile the safety culture in the researched environment. 

 The analysis of qualitative information, based on discussions of the FG and PW technique, was guided by a thematic content analysis, consisting of pre-analysis, exploration of the material, and treatment of obtained results and interpretations ^(^ ^18^ ^)^ . Pre-analysis corresponds to the researcher’s first contacts with the material. Once in possession of the set of information, it was organized in order to respond to the objectives, and a floating and exhaustive reading was carried out, so that the researcher could establish greater contact with the text. The exploration of the material is the raw data coding phase, in which the core meaning of the text was sought by separating words, sentences or paragraphs, which were classified and aggregated into categories. Finally, in the treatment of obtained results and interpretations, the raw results were treated in a meaningful and valid way. At this point, through reliable results, the information obtained was analyzed, giving interpretations and purposes to the objectives seen previously ^(^ ^18^ ^)^ . 

An interface between qualitative and quantitative findings allows for a more complete and in-depth analysis of the occurrences in question, making it possible to find patterns, obtain insights and a more comprehensive understanding. Furthermore, the literal transcriptions, narratives and photos were organized into files using the NVivo 11 program.

 Data integration occurred based on the sequential explanatory approach, allowing the connection between quantitative and qualitative elements, in order to complement each other and allow a holistic understanding of the phenomenon under study, and verify whether or not the qualitative data converged with the quantitative data ^(^ ^19^ ^)^ . For data complementarity, inferences obtained from the FG were explored. This integration was made possible through a joint-display, an enlightening approach to demonstrate QUAN→Qual integration that visually represents integration in mixed methods designs ^(^ ^20^ ^)^ . The integrated data were those referred to in the focus groups with direct adherence to one or more domains of the SAQ/SC, and which guided the first stage of the study. In this way, integration occurred in a connected and inductive way. 

## Ethical aspects

The study met ethical and legal aspects, highlighting that all participants were informed about the implications of the research when signing the Free and Informed Consent Term, and were identified with the letter P followed by the assigned participation number, FG, date and category, in order to guarantee anonymity. Photographs are presented to illustrate highlights of the research. The project was approved by the Research Ethics Committee of the study institution under opinion number 4.092.333/2020 and Certificate of Presentation of Ethical Appreciation 31032220.9.0000.5335.

## Results

 The data from the quantitative phase, referring to the characterization of health and support areas professionals who work in the institution’s five SC groupings, obtained through the SAQ/SC, are presented in Table 1 . 

**Table 1:** - Characterization of health and support areas professionals working in the SC based on the SAQ/SC ^*^ regarding the variables gender, age, ethnicity, professional category, professional experience, working time, working regime and shift. Porto Alegre, RS, Brazil, 2021

**Variables**	**N** ^†^ **= 172**
**Gender (%)** ^‡^	
Female	124 (74.7)
Male	42 (25.3)
**Age (years)** ^§^	36.8 ± 8.8
**Ethnicity (%)** ^‡^	
White	126 (7.3)
Black	18 (11.0)
Brown	12 (7.2)
Afro-descendant	9 (5.5)
**Professional category (%)** ^‡^	
Scrub or circulating	100 (58.4)
Surgical Center Nurse	22 (12.8)
Surgeon/assistant surgeon	16 (9.3)
Surgery resident or intern	9 (5.3)
Head nurse	7 (4.0)
Administrative	6 (3.5)
Anesthesiologist	5 (2.9)
Perfusionist	3 (1.6)
Anesthesiology resident	1 (0.6)
Others	3 (1.6)
**Professional experience (years)** ^||^	7.0 (3.0 – 14)
**Working time (years)** ^||^	5.0 (2.0 – 10)
**Working regime (%)** ^‡^	
Part time (36 h)	76 (47.2)
Full time (40 h)	51 (31.7)
Hired	28 (17.4)
Cooperative	5 (3.1)
Other	1 (0.6)
**Shift (%)** ^‡^	
Part time	82 (54.7)
Full time	44 (29.3)
Variable	14 (9.3)
Night	10 (6.7)

* SAQ/SC = Safety Attitudes Questionnaire/Surgical Center;

† N = Absolute number;

§ Mean ± standard deviation;

|| Median (25-75 percentiles);

‡ Absolute number (percentage)

The first part of the instrument, which addresses the Quality of Communication and Collaboration experienced among professionals during the work routine at the SC, demonstrates that only the nursing category (nurse, scrub and circulating) reaches the minimum score (≥75), reflecting a positive perception of safety culture. The average score for SC nurses is 78.9 (SD 24.6), and for head nurses, 79.9 (SD 24.9). Among scrub and circulating, the mean remains at 78.5 (SD 22.8). In the global analysis, there is a significant difference between the scores attributed by these nursing professionals and other health workers, such as surgery residents and interns (60.6; SD 29.6; p= 0.043); perfusionists (28.7; SD 38.5; p < 0.001); and anesthesiology residents and interns (51.4; SD 32.8; p= 0.019).

The second part of the instrument, which encompasses the six domains of the SAQ/SC, presents a median variation of 70.0 (61.2-85.0) to 81.2 (68.7-87.5) points per domain, with an overall median of 77.6 (63.1-88.8). The SC named “F” exhibited, in general, the lowest score: 71.9 (61.7-85.9). Also in this location, the lowest scores with significant differences in relation to the others were identified for Management Perception 62.5 (52.5-75.0; p=0.016), followed by Working Condition 68.7 (54.1-87.5; p=0.001). Furthermore, in the global analysis, it is noteworthy that the domains Safety Climate 78.5 (64.2-85.7; p=0.047), Management Perception 70.0 (61.2-85.0; p=0.016) and Working Condition 68.7 (54.1-87.5; p=0.001) present statistically significant differences between the five grouped SC.

 The qualitative results obtained through the FG technique and photographic means, from the perspective of ecological and restorative thinking, are grouped into two categories: (1) *Understanding the safety culture in SC* and (2) *Communication as a resource for building a safety culture* . 

In category 1, the ideas brought by participants in the FG meetings about what safety culture is and its importance for patients and healthcare teams are presented. During the debates, the need to explain to patients the importance of the Surgical and Anesthesia Consent Form was discussed, as well as the correct completion of the safe surgery checklist by professionals, highlighting that this tool reduces errors in surgeries and contributes to safe practices.

 The participants’ statements highlight that a strengthened safety culture contributes to a favorable work climate, as illustrated in the excerpts: *They understand and know the importance of the tools [safe surgery checklist], they only skip steps due to the rush, mistakes are not alone, there are several steps not carried out. The obvious is not obvious (P6, FG 1, 11/06/2020). Medical teams resistant to protocols [completion of surgical and anesthesia consent terms] (P4, FG 1, 11/16/2020).*

**Figure 1:**
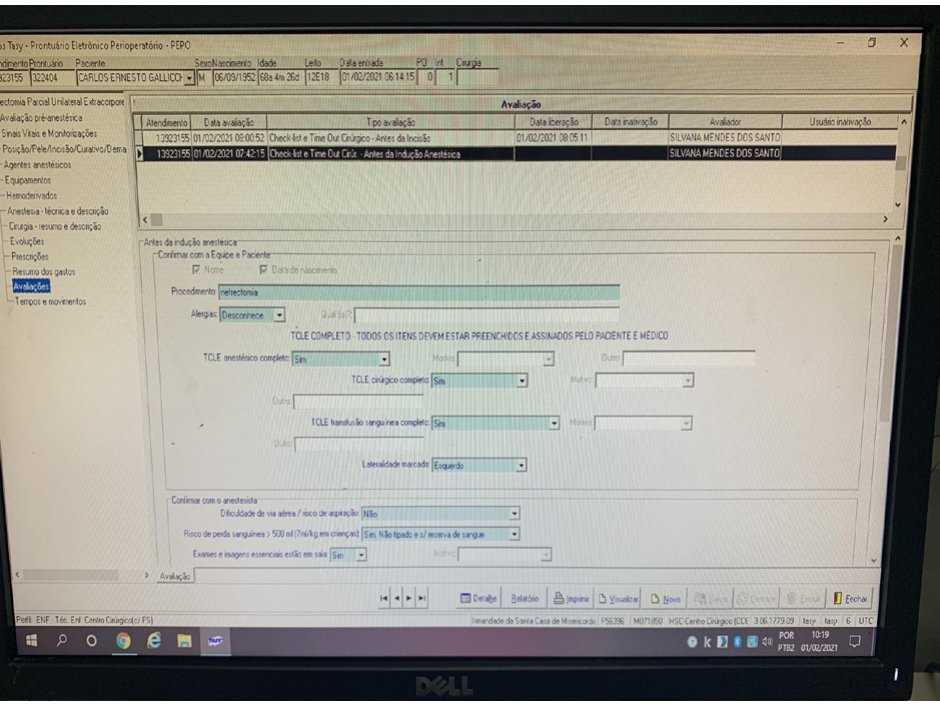
- Authorial photo of the use of the safe surgery checklist

Figure 1 shows the safe surgery checklist at the investigated institution, which is completed in the system in stages: before anesthetic induction with the patient’s participation, before the surgical incision and before the patient leaves the operating room. 

**Figure 2:**
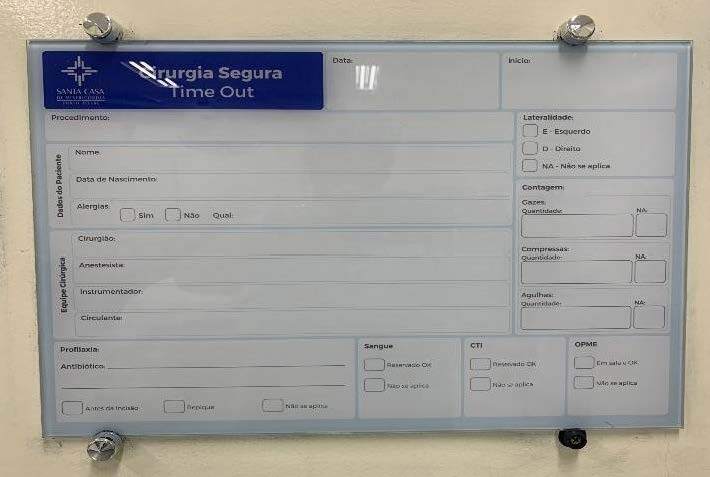
- Authorial photo of the checklist information board placed in the operating rooms

 In addition, a board is displayed in the operating rooms with the most important information to be filled out for each surgical procedure, which guides the completion of the time out (surgical pause) and sign out (before the patient leaves the operating room) steps, as shown in Figure 2 . 

There are initiatives by the institution to value the principles of safety through the implementation of boards for the safe surgery checklist and panels to disseminate institutional and sectoral information. Such aspects are addressed in the FG and PW.

The participants highlight that many medical teams refuse to apply the surgical and anesthetic consent terms, alleging lack of time. This same argument is used to not carry out the safe surgery checklist, although, according to nursing professionals, this procedure takes two or three minutes.

Category 2 addresses the issues of accessibility of managers, shared information and empathy. It is emphasized that communication between medical and nursing teams occurs in a calm manner, and that daily interaction between workers facilitates the communication process.

 In the FG, the participants confirm this weakness, not identifying a present and active leadership in the SC that have lower scores. On the other hand, in the discussions, SC professionals with positive scores highlight the accessibility of the sector’s leadership and professional recognition as important elements for teamwork, as in the following statements: *[...] some do not have access to resolve issues, there should be more meetings to explain what the group wants to talk about, that is needed (P4, FG 1, 11/06/2020). I agree with more meetings (P1, FG 2, 11/06/2020).*

 Insufficient staffing is also mentioned in group discussions, linked to the institution’s high turnover, due to lack of recognition and retention of professionals. Employees express that they feel overwhelmed and exhausted, a feeling they verbalize: *Lack of responsibility of doctors, anesthetists, improve nursing dimensioning, reduce absenteeism (P6, FG 1, 11/06/2020). Resistance from doctors in the processes (P4, FG 1, 11/06/2020). They don’t respect [doctors] (P1, FG 1, 11/06/2020). [...] They are leaving due to lack of team, collegiality, we are losing many good people, lack of recognition, motivation. The bad is praised and the good is demotivated (P4, FG 2, 11/06/2020). People feel overwhelmed, people are exhausted, tired (P2, FG 2, 11/06/2020).*

In this section of reports, in addition to overload and exhaustion, there is a perception of little engagement from other categories, such as doctors, which deserves to be highlighted. Furthermore, it is observed that feelings related to the lack of recognition and motivation have an important influence on dedication and execution of work. The latter have an intrinsic connection with the role of nursing leadership, and, normally, improvements do not generate costs, so it would be opportune to explore this aspect.

**Figure 3:**
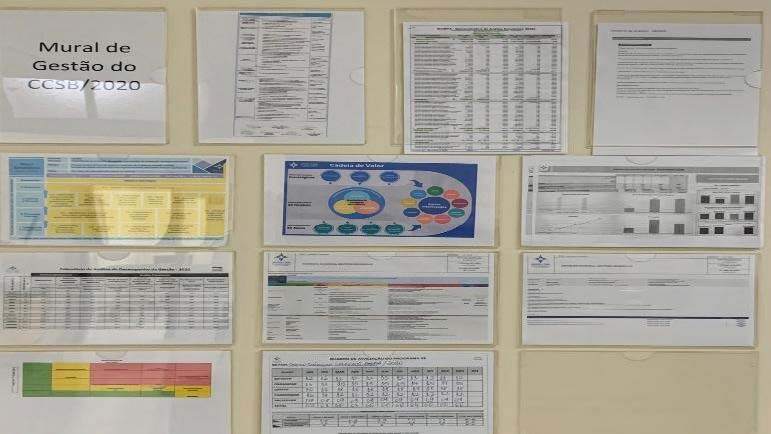
- Authorial photo of the information panel

 They also report missing meetings with teams as a strategy to share information among health professionals and contribute to patient safety. Among the actions implemented and highlighted in the PW is the use of information panels, which aims to facilitate communication ( Figure 3 ). Participants cite the panels in the hall way and rest spaces as an important resource for transmitting institutional information, such as economic-financial results, quality indicators and monthly adverse events, according to the statements: *I think it communicates a lot [more], it’s important that [professionals] know the goals (P1, FG 2, 02/11/2021). They [professionals] like to know the results (P2, FG 2, 02/11/2021). We need to get closer to the technicians, review the goals, what we achieved in the month (P1, FG 2, 02/11/2021).*

 The excerpts from the participants’ statements that support the thematic categories are presented in the joint display ( Figure 4 ) in an integrated way with the quantitative data, and seek to contribute to the understanding of the results obtained by the SAQ/SC. 

**Figure 4:** - Integration of quantitative and qualitative results through joint display

**Quantitative results**	**Qualitative results**	**Integration**
Domain 1: Safety Climate – in the general analysis it obtains a score above 78.5, denoting a positive safety culture. There is a statistically significant difference between the five grouped SC ^†^ (p=0.047). SC ^†^ F ^‡^ obtains a negative score (71.4), and SC ^†^ G ^‡^ has a borderline score of 75.0. The rest are above the cutoff point.	Strategies such as reformulating the checklist are discussed in the FG ^*^ , making it easier to fill in information and avoiding wasting time on irrelevant elements.	Progress towards strengthening a safety culture was observed in the actions described in the FG ^*^ and in the photographic walks, highlighting improvements in information on the panels, as well as the implementation of boards with the safe surgery checklist. These strategies are seen as a resource to disseminate the patient safety culture in the institution, and help to explain the positive perception obtained by the scores in most SC ^†^ in the Safety Climate domain.
Domain 2: Management Perception – in the global analysis, it is one of the domains with the lowest score (median 70) among the SC ^†^ . SC ^†^ F ^‡^ , G ^‡^ and H ^‡^ present weaknesses with scores below 75 points. SC ^†^ grouped A+B ^‡^ and C+D+E ^‡^ present positive perceptions. When comparing the groups, it was found that SC ^†^ F ^‡^ has the lowest median (62.5), with a significant difference (p=0.016) in relation to SC ^†^ A+B ^‡^ (80) and C+D+E ^‡^ (80).	This domain addresses the approval or disapproval of leadership actions related to safety issues.	Management Perception presents weaknesses in most SC ^†^ in both stages. The leadership profile that is not very present and active is a prominent aspect in the SC ^†^ with lower scores. This domain can be improved through individual conversations, feedback and alignment meetings with managers. Such aspect was highlighted by the FG ^*^ participants (group B) when they mentioned not identifying the presence of the manager in the care environment.
Domain 4: Working Condition – presents a score of 68.7 among the SC ^†^ . In the overall analysis, it is the domain with the lowest score, indicating greater weaknesses in the construction of a safety culture in the SC ^†^ evaluated. Only SC ^†^ G ^‡^ presents a favorable score (83.3), with a significant difference (p=0.001) in relation to SC ^†^ A+B ^‡^ and F ^‡^ , both with 66.6. The remaining SC ^†^ have borderline scores or below the cutoff point of 75.	This domain is related to professionals’ perception of the quality of the work environment. In the FG ^*^ work overload is signaled, linked to the intense dynamics in the day-to-day life of the SC ^†^ . Also, the importance of assistance among professionals is highlighted.	The Working Condition presents a negative perception among the SC ^†^ in the quantitative stage, a fact reinforced in the participants’ statements. The lack of personnel and intense work dynamics result in overload for workers. There are opportunities for improvement through resizing teams, encouraging collaboration between peers and reviewing daily activity schedules.
Domain 5: Communication in the Surgical Environment – has better scores overall and in all SC ^†^ , and demonstrates a positive perception regarding the quality of communication and collaboration in the multidisciplinary team. In the overall score, it has a median of 81.2. Only SC ^†^ F ^‡^ presented a borderline score, 75.0, with no significant difference between the groups (p=0.101).	Participants highlight the importance of interaction between healthcare teams as fundamental to daily work, along with communication.	Despite the positive score in the quantitative stage, the need for a greater number of meetings and interaction between health teams is highlighted in the qualitative stage. Even though it is identified that the use of WhatsApp contributes to the agility of communication between teams, spaces for exchange and dialogue can improve communication and strengthen the safety culture.

* FG = Focus group;

† SC = Surgical Center;

‡ Designation letters of each of the surgical centers researched

Domains 3 (Stress Perception) and 6 (Perception of Professional Performance) in the quantitative stage present positive scores. Domain 3 has the highest score in the global assessment (81.2), while domain 6 has a median of 75.0 in the global analysis. SC A+B and G have better scores (81.2). The rest have borderline scores. As these topics were not discussed in the FG, these domains were not included in the joint display for data integration.

## Discussion

 The data characterizing the sample in the present study are consistent with other findings in the Brazilian context, indicating a predominance of women (74.7%), nursing professionals 126 (75.2%), with a median age of 37 years, working in SC. A survey carried out in Brazil in a SC of a university hospital, in order to assess the risk of pathogenic diseases in these workers, showed that the majority of this sector are women (81.9%) aged between 36 and 50 years, corroborating the findings of this study ^(^ ^21^ ^)^ . 

 The first part of the SAQ/SC instrument demonstrated that only the nursing category (nurse, scrub and circulating) reached the minimum score (≥75) to be considered a positive perception of safety culture. In another study ^(^ ^22^ ^)^ , nurses also obtained higher average scores in items related to the quality of communication and collaboration, similar to the results of this study. A study carried out in the SC of a public university hospital in the state of Paraná, which found a lower score among scrub/circulating staff, suggests that there is a low quality of communication and collaboration in the other categories that work in the SC ^(^ ^21^ ^)^ . 

 Adequate communication and collaboration among SC team members are fundamental to ensuring patient safety during surgery, so everyone has an important role to play, and it is essential that each one understands each role and works together ^(^ ^22^ ^)^ . 

 Effective communication between healthcare professionals, patients and families is a crucial factor in providing quality and safe care. It is the basis for shared decision-making, early identification of problems and resolution of conflicts, all fundamental elements of patient-centered care. From this perspective, nurses play a prominent role in integrating information between teams, reducing the chances of adverse events and promoting open and honest communication between team members ^(^ ^23^ ^-^ ^24^ ^)^ . 

 When a safety culture is established in an institution, communication between professionals becomes easier and more effective. This is because there is greater trust among team members, who feel more comfortable communicating openly and sharing relevant information. Furthermore, when all team members are aware of the safety measures adopted and understand the importance of following them, teamwork and collaboration are improved, which contributes to the provision of quality patient care ^(^ ^25^ ^)^ . 

The general descriptive analysis of the SC grouping score, based on the SAQ/SC domains, revealed a favorable perception of the safety climate in the locations considered. This indicates a positive perception regarding patient safety in these environments. Among the SAQ/SC domains, Management Perception and Working Condition stood out as the most fragile in the SC evaluated. This suggests that employees in these centers may have a less positive perception about management and existing working conditions. There may be a need for improvements in these aspects to promote a stronger safety culture.

On the other hand, the domains Communication in the Surgical Environment, Safety Climate, Perception of Professional Performance and Stress Perception presented favorable scores. This indicates that SC employees have a positive perception in these aspects, which suggests the existence of a more robust safety culture and good communication between the professionals involved.

 The integrated analysis of the results reinforces the weakness of the Management Perception and Working Condition domains in the evaluated SC, both due to the negative scores and the professionals’ statements in the focus groups. It is interesting to note that similar results were also found in other studies regarding management perceptions and working conditions in SC. This consistency in the results suggests that the low management perception, together with unfavorable working conditions, can lead health professionals to be unaware of the institutions’ management commitment to the hidden factors of safety culture. Consequently, this lack of knowledge can be reflected in negative scores and indicate a distance between the team and their superiors ^(^ ^26^ ^-^ ^27^ ^)^ . 

 To promote the development of a culture of patient safety, it is necessary to invest and develop training and engage health professionals with this topic. Furthermore, it is essential that hospitals provide the necessary support resources for healthcare professionals ^(^ ^28^ ^)^ . 

The positive score in the Safety Climate domain was reinforced by the participants’ statements in the qualitative stage, highlighting the importance of a strengthened safety culture to create a favorable work climate.

 It is interesting to note that a study carried out in three public and private hospitals in the state of Rio Grande do Sul compared the safety climate of these institutions, and identified positive scores in the private service, while philanthropic institutions presented scores below average, highlighting a weakness in the safety culture in these institutions ^(^ ^29^ ^)^ . 

These findings highlight the importance of a strong safety culture to promote a positive and safe work climate in SC. An established safety culture is essential to encourage open communication, continuous learning and engagement by all healthcare professionals in identifying and preventing adverse events.

 The Stress Perception domain also presented an above score in another study, indicating that participants perceive stressful factors in the work environment ^(^ ^30^ ^)^ . This perception may be especially relevant for professionals who work in SC, since they are exposed to a series of stresses due to the challenging work environment. 

 Professionals who work in SC face high levels of stress due to several factors, such as closed environment, risks involved, different routines, need for technical skills and high productivity required ^(^ ^31^ ^)^ . These elements can contribute to the emotional and psychological pressure faced by surgical professionals. 

 Given this scenario, it is suggested to reorganize activities and resize teams as measures to improve quality of life at work and reinforce patient safety in the surgical environment ^(^ ^26^ ^)^ . By reevaluating the distribution of tasks and workload, it is possible to promote a healthier balance for professionals, reducing stress and increasing well-being at work. 

The borderline score in the Perception of Professional Performance domain indicates a neutral assessment by the surgical team in this aspect, according to the results of the SAQ/SC. This domain is related to the way in which fatigue and work overload affect individuals’ professional performance.

 Professional performance is intrinsically linked to job satisfaction, reflecting the individual experience of each professional. Job satisfaction is defined as a positive feeling regarding work, which encompasses aspects such as work content, development opportunities, recognition, working conditions and relationships with colleagues and superiors ^(^ ^30^ ^)^ . 

It is important to consider that job satisfaction and professional performance are affected by a variety of factors, including working conditions, organizational support, recognition and growth opportunities. Therefore, it is essential that healthcare institutions are aware of these aspects and adopt measures to promote a healthy and balanced work environment, which values the well-being of professionals.

 Job satisfaction not only affects the well-being and health of professionals, but is also related to the prevention of occupational diseases and the achievement of better results in the work process, which includes the activities performed by nurses and other health team members ^(^ ^31^ ^)^ . 

When professionals are satisfied at work, they tend to be more engaged, motivated and dedicated to their responsibilities. This can lead to greater efficiency, productivity and quality of patient care. On the other hand, job dissatisfaction can lead to a negative environment, demotivation, increased stress and possible errors or failures in providing care.

As limitations of the study, the reduced number of respondents in some professional categories stands out, due to the critical health period caused by the Covid-19 pandemic and the significant reduction in surgical schedules. The absence of representatives in the FG from all categories investigated in the first stage, including doctors, perfusionists and pharmacy or hygiene assistants, is a limitation. However, the study offers relevant reflections on the climate and safety attitudes in the SC, and can be considered to provide assertive guidance for safe care.

The study results indicate a better perception of the safety climate by the nursing team, which signals a greater potential for their engagement in building a safety culture. In this sense, the need for a more in-depth assessment of strategies for engaging medical teams stands out, with the aim of promoting interprofessional and collaborative work.

The contributions of this study to care practice include highlighting the importance of developing professionals who occupy management positions, seeking a greater approximation and understanding of the needs of the care team. This implies a more assertive and sensitive approach to promoting a healthy and safe work environment.

Furthermore, the study highlights the need to invest in the working conditions offered to employees. This involves adjusting the workload, considering an equitable distribution of tasks, and reviewing staff sizing. By providing an adequate workload, surgical centers can promote a more positive safety climate, reducing the risk of errors and improving the quality of care provided. These suggestions aim to improve the quality of care, promoting a more collaborative, safe and effective work environment for the entire care team.

## Conclusion

The SC studied presented results that indicate a positive perception of the safety climate among health and support professionals. The domains Stress Perception, Communication in the Surgical Environment, Safety Climate and Perception of Professional Performance obtained higher scores in relation to safety culture, a finding that was reinforced in the qualitative analysis.

The analysis carried out in this survey regarding safety attitudes in SC provided an in-depth understanding of the challenges faced in this complex environment. The combination of quantitative and qualitative data, which is an approach still little explored in perioperative nursing, proved to be challenging, but prolific.

Although the global analysis of the domain Communication and Collaboration between Teams was positive, both quantitative and qualitative data identified opportunities for improvements in this process. The nursing team seems to adhere more easily to institutional processes and routines aimed at patient safety, which reflects greater cultural strengthening of this category with regard to safe care.
