# Supplementary material for: Challenges of safety culture in Surgical Center: mixed methods study
Source: Rev Lat Am Enfermagem. 2024 Jul 29;32:e4206. doi: 10.1590/1518-8345.7001.4206 (PMC11295263; doi:10.1590/1518-8345.7001.4206)
Supplement: Translation

# Translation: Desafíos de la cultura de seguridad en Centro Quirúrgico: estudio de métodos mixtos ^*^

**DOI:** 10.1590/1518-8345.7001.4205

**Authors:** Nery José de Oliveira Junior, Caren de Oliveira Riboldi, Daniela Campos de Andrade Lourenção, Vanessa de Brito Poveda, João Lucas Campos de Oliveira, Ana Maria Müller de Magalhães

**Affiliations:** 1 Universidade Federal do Rio Grande do Sul, Escola de Enfermagem, Porto Alegre, RS, Brasil.; 2 Universidade de São Paulo, Escola de Enfermagem, São Paulo, SP, Brasil.

**Keywords:** Seguridad del Paciente, Cultura Organizacional, Enfermería Perioperatoria, Centros Quirúrgicos, Enfermería, Ambiente de Instituciones de Salud

## Abstract

**Objetivo:**

analizar las actitudes de seguridad de los profesionales de la salud y de áreas de apoyo que trabajan en el Centro Quirúrgico.

**Método:**

estudio explicativo secuencial de métodos mixtos. La etapa cuantitativa abarcó a 172 profesionales de la salud y de apoyo en ocho Centros Quirúrgicos de un complejo hospitalario. Se aplicó el Cuestionario de Actitudes de Seguridad/Centro Quirúrgico. En la etapa cualitativa posterior, participaron del Grupo Focal 16 profesionales. Se utilizaron métodos fotográficos desde la perspectiva del pensamiento ecológico y restaurativo, y el análisis de datos se produjo de manera integrada, por medio de conexión.

**Resultados:**

el puntaje general, por grupo de Centros Quirúrgicos, con base en los dominios del Cuestionario de Actitudes de Seguridad/Centro Quirúrgico, revela una percepción favorable del clima de seguridad, con énfasis en los dominios Percepción del Estrés, Comunicación en el Ambiente Quirúrgico, Clima de Seguridad y Percepción del Desempeño Profesional. El análisis global del dominio Comunicación y Colaboración entre Equipos parece positivo y está corroborado por datos de la etapa cualitativa, que resalta la importancia de la interacción y de la comunicación entre los equipos de salud como fundamentales para el trabajo diario.

**Conclusión:**

la percepción de las actitudes de seguridad entre los profesionales de la salud y de apoyo fue positiva. Se destaca la percepción del equipo de enfermería como más cercana o favorable a actitudes coherentes con la cultura de seguridad.

## Destacados

**(1)** La comunicación y el trabajo en equipo refuerzan la cultura de seguridad.

**(2)** Equipo de enfermería más comprometido con la cultura de seguridad.

**(3)** Percepción favorable del clima de seguridad por parte de los profesionales de la salud y de apoyo.

**(4)** Adhesión del equipo de enfermería a los procesos y rutinas institucionales.

## Introducción

 La Organización Mundial de la Salud destaca que cada año, en países de ingresos bajos y medios, ocurren aproximadamente 134 millones de eventos adversos y 2,6 millones de muertes asociadas a entornos inseguros, lo que demuestra que la seguridad del paciente debe ser un objetivo permanente. En este contexto, si bien existen avances en la evidencia sobre el impacto de los eventos adversos, las medidas para reducir, mitigar, prevenir y medir los riesgos potenciales en términos de tecnología, recursos financieros e intervenciones aún son incipientes ^(^ ^1^ ^)^ . Para implementar mejoras es necesario fomentar una cultura que favorezca la seguridad del paciente en las organizaciones de salud ^(^ ^2^ ^)^ . 

 La cultura de seguridad se entiende como un conjunto de valores y prácticas individuales y colectivas enfocadas a reducir los riesgos y daños al paciente ^(^ ^3^ ^)^ . Al tratarse de un elemento cultural y eminentemente polifacético, que emana de una concepción organizacional, existen factores que se consideran medibles ^(^ ^4^ ^)^ . Un ejemplo es el clima de seguridad, que se refiere a la percepción de los actores organizacionales sobre políticas y prácticas (in)seguras en el ambiente del cuidado en salud ^(^ ^3^ ^-^ ^4^ ^)^ . 

 Los medios para analizar la cultura y el clima de seguridad incluyen cuestionarios, entrevistas y observaciones, o incluso evaluaciones externas por parte de expertos en la materia. Dichos análisis permiten verificar el nivel de compromiso institucional y profesional con valores, creencias, recursos, actitudes y comportamientos relacionados con la seguridad del paciente ^(^ ^1^ ^,^ ^4^ ^-^ ^5^ ^)^ . En otras palabras, si bien en la práctica el clima de seguridad se evalúa por medio de la dinámica de trabajo, incluyendo principalmente la percepción de los trabajadores, esto será un reflejo de cómo se posiciona la cultura organizacional en relación al cuidado seguro ^(^ ^2^ ^,^ ^5^ ^)^ . 

 Las investigaciones relacionadas con la cultura de seguridad en el ambiente hospitalario están en constante crecimiento ^(^ ^6^ ^-^ ^7^ ^)^ , lo que refuerza el hecho de que los valores organizacionales impactan directamente en los resultados de seguridad, abarcando también la ocurrencia de eventos adversos, es decir, daños concretos al paciente ^(^ ^8^ ^)^ . Tales valores deben fortalecerse en cualquier entorno de prestación de atención, aunque se reconoce que algunos ambientes tienen un riesgo mayor o más evidente debido a la naturaleza de los procesos de trabajo desarrollados, como el Centro Quirúrgico (CQ) ^(^ ^9^ ^)^ . 

 Los llamados *near misses* o *casi errores* ya fueron menos frecuentes en los CQ que en otras unidades, como hospitalización, cuidados intensivos y pediatría ^(^ ^10^ ^)^ . Por otro lado, resulta alarmante la aparición de eventos adversos de muy alta gravedad – conocidos como *never events* –, en el intraoperatorio y postoperatorio inmediato, como cirugía en lateralidad incorrecta, procedimiento y/o paciente incorrectos, retención de material en el paciente y quemaduras por electrocauterización ^(^ ^11^ ^)^ . A esto se suma el hecho de que hasta el 90% de los eventos adversos relacionados con la cirugía se clasifican como prevenibles ^(^ ^12^ ^)^ , por lo que es imperativo instituir y fomentar medidas de seguridad del paciente en el CQ, lo que se puede lograr con mayor asertividad por medio de análisis sistemáticos de las actitudes de seguridad en esos entornos. 

Teniendo en cuenta lo anterior, esta investigación se basó en las siguientes preguntas orientadoras: ¿Cuál es la percepción del clima de seguridad entre los profesionales de la salud y de áreas de apoyo que trabajan en CQ? ¿Qué aspectos impactan la cultura de seguridad en este escenario? Por lo tanto, el objetivo de este trabajo fue analizar las actitudes de seguridad entre los profesionales de la salud y de áreas de apoyo que trabajan en CQ.

## Método

### Diseño del estudio

 Se trata de un estudio de métodos mixtos, anclado en el diseño explicativo secuencial, que combina elementos cuantitativos y cualitativos para responder a las preguntas de investigación de una manera más amplia y completa. La primera fase, con un enfoque cuantitativo y de mayor peso (CUAN), permitió medir variables específicas de manera objetiva, mientras que la etapa posterior, con un enfoque cualitativo secundario (Cual), tuvo como objetivo profundizar en la comprensión del fenómeno previamente medido. El análisis inicial de los resultados cuantitativos guio la recopilación de datos cualitativos (CUAN→Cual), apoyando la exploración de insights con mayor detalle y facilitando el descubrimiento de nuevos significados, interpretaciones y relaciones entre las variables ^(^ ^13^ ^)^ . 

### Escenario de recolección de datos

El estudio se realizó en un único complejo compuesto por ocho hospitales, ubicado en la ciudad de Porto Alegre-RS, Brasil. Cada local cuenta con un CQ, totalizando 53 salas dedicadas a cirugías generales y especializadas. En total, la productividad quirúrgica es de aproximadamente 6.000 procedimientos por mes. Los CQ incluidos en esta investigación se caracterizan de la siguiente manera: CQ “A” (13 salas), que realiza cirugías de diferentes especialidades; CQ “B” (4 salas), destinado a procedimientos oftalmológicos; CQ “C” (3 salas), que realiza cirugías de especialidad en neumología; CQ “D” (3 salas), que realiza cirugías neurológicas; CQ “E” (4 salas), que realiza cirugías cardíacas; CQ “F” (7 salas), que atiende la especialidad de oncología; CQ “G” (12 salas), que cubre las especialidades de cirugía plástica y trasplantes; y CQ “H” (7 salas), que atiende la especialidad de pediatría.

## Período

El estudio se llevó a cabo entre junio de 2020 y febrero de 2021.

### Población

La población de estudio estuvo compuesta por profesionales de la salud y de áreas de apoyo de los ocho CQ. Los profesionales de la salud fueron: cirujanos y anestesiólogos, médicos residentes, enfermeras y técnicos de enfermería. Como áreas de apoyo se consideraron auxiliares administrativos, auxiliares de farmacia e higiene.

### Criterios de selección

Los criterios de elegibilidad para participar en el estudio consistieron en ser parte del equipo de uno de los CQ y no estar de vacaciones o licencia funcional durante el transcurso de la recolección de datos.

### Participantes

La etapa cuantitativa abarcó a 172 profesionales, distribuidos entre las siguientes categorías: instrumentador quirúrgico y circulante (100), enfermero de CQ (22), cirujano o cirujano asistente (16), residente de cirugía o interno (9), jefe de enfermería (7), asistente administrativo (6), anestesiólogo (5), perfusionista (3), residente de anestesiología (1) y auxiliares de farmacia o higiene (3). Cabe señalar que hubo un bajo número de cirugías realizadas debido a las restricciones durante el período de pandemia de Covid-19, lo que se tradujo en una menor presencia de equipos médicos, al reducirse los horarios quirúrgicos. Estas restricciones también impactaron la siguiente etapa, cualitativa, que contó con 16 participantes entre: enfermeros (5), técnicos de enfermería (10) y auxiliar administrativo (1). Para esta fase, además de participar en la etapa anterior, era necesario trabajar en los CQ que presentaron puntajes más favorables o desfavorables en la etapa cuantitativa. En el primer encuentro participaron ocho representantes de cada CQ, y en el segundo participaron 12 trabajadores, seis de cada grupo.

### Instrumentos utilizados para recolectar la información

En la etapa cuantitativa se utilizó el Cuestionario de Actitudes de Seguridad/Centro Quirúrgico (CAS/CQ) con el objetivo de medir el clima de seguridad en los servicios de salud a partir de la percepción de los profesionales sobre la seguridad del paciente. Los investigadores optaron por una versión menos actualizada del instrumento, porque culturalmente, en el lugar donde se realizó el estudio, los técnicos de enfermería desempeñan alternativamente el papel de circulante o instrumentador en el horario de trabajo. En la versión actual del instrumento, estas funciones fueron agrupadas, no aplicándose a la realidad en cuestión.

 La versión brasileña del CAS/CQ consta de una escala tipo Likert y está dividida en tres partes. La primera, con 15 afirmaciones, se refiere a la calidad de la comunicación y colaboración entre los profesionales que actúan en el ámbito quirúrgico, a la que el participante de la investigación debe responder sobre su relación con cada una de las categorías profesionales. El segundo está compuesto por 40 enunciados, divididos conceptualmente en seis dominios: clima de seguridad (siete ítems), percepción de la gestión (cinco ítems), percepción del estrés (cuatro ítems), condición de trabajo (seis ítems), comunicación en el ambiente quirúrgico (cuatro ítems) y percepción del desempeño profesional (cuatro ítems). La tercera parte cubre información demográfica (género, raza/etnia, categoría profesional, tiempo de experiencia, turno de trabajo, entre otros) y un espacio donde el participante puede escribir tres recomendaciones para mejorar la seguridad del paciente en CQ, e indicar si ya había respondido el instrumento previamente ^(^ ^14^ ^)^ . 

 La etapa cualitativa posterior utilizó la técnica de Grupo Focal (GF) y métodos de investigación fotográfica desde la perspectiva del pensamiento ecológico y restaurativo, por medio de caminata fotográfica (CF) ^(^ ^15^ ^-^ ^16^ ^)^ . En esta etapa, la muestra fue intencional y por conveniencia, entre quienes participaron en la primera etapa (cuantitativa). 

Para la realización de los GF se utilizó un guión elaborado por los investigadores, abarcando los temas: cultura de seguridad, qué influye en la cultura de seguridad, relación del equipo multidisciplinario en el ambiente del CQ y clima de seguridad en el CQ.

La técnica de GF se organizó y condujo de acuerdo con el siguiente guión: apertura de la sesión, integración de los participantes, explicación de la dinámica de las discusiones, configuración del grupo, debate, síntesis de los momentos previos y cierre del encuentro. Además, se firmó un acuerdo de confidencialidad, reforzando que los debates e ideas discutidas en los encuentros estarían restringidos a los miembros del grupo.

El guión fue planificado en base a la necesidad de complementar/profundizar los hallazgos cuantitativos. Por lo tanto, la investigación de estos elementos fue importante para constituir una base de datos cualitativos, creando un repositorio de información rica y contextual que puede ser verificada, para encontrar inferencias y aclaraciones sobre los hallazgos cuantitativos.

### Recolección de datos

En la primera fase del estudio, de recolección de datos cuantitativos, se aplicó el cuestionario en forma impresa y presencial, durante la jornada laboral de los profesionales. Se siguió la selección de la muestra por conveniencia, de junio a julio de 2020. La segunda fase de la investigación, de carácter cualitativo, se desarrolló entre octubre de 2020 y febrero de 2021. La muestra en esta etapa fue intencional y por conveniencia, seleccionada entre quienes participaron en la primera etapa (cuantitativa).

Se puso a disposición de quienes estaban interesados en participar en el GF y en la CF en el CQ una lista definida según los mejores y peores resultados del cuestionario CAS/CQ. Se seleccionaron los primeros 12 inscriptos de cada GF y se realizaron cuatro encuentros, dos con los profesionales del CQ con mejor puntuación (grupo A) y dos con los vinculados al lugar con peor puntuación (grupo B).

El primer encuentro del GF tuvo como objetivo discutir temas de interés, relacionados con aspectos de la cultura de seguridad y con el relevamiento de temas considerados prioritarios por los participantes, con el fin de componer la hoja de ruta de la CF. Después del primer GF, los temas tratados fueron validados por un miembro de cada grupo (enfermera), quien posteriormente acompañó la CF por el CQ. El segundo encuentro tuvo como foco principal la discusión de las fotografías obtenidas, caracterizándose como GF para foto-elicitación.

### Procesamiento y análisis de datos

 Para fines de análisis, se ordenaron los CQ en cinco grupos (A+B; C+D+E; F; G; H), según el número total de participantes, similitud de procesos, número de quirófanos y volumen de procedimientos. Los datos cuantitativos de la primera etapa fueron analizados mediante el *software Statistical Package for the Social Sciences* versión 21.0. Se utilizó estadística descriptiva para caracterizar la muestra, mediante la cual las variables discretas se presentaron en forma de media y desviación estándar o mediana y percentil, y las variables categóricas se expresaron en forma de frecuencia absoluta y relativa. 

 Para el análisis estadístico, se consideraron como variables dependientes los puntajes de los dominios CAS/CQ: Clima de Seguridad, Percepción de la Gestión, Percepción del Estrés, Condición de Trabajo, Comunicación en el Ambiente Quirúrgico y Percepción del Desempeño Profesional; y como variables independientes: edad, género, profesión y tiempo de experiencia en la especialidad. Se utilizaron las pruebas de Chi-Cuadrado y Shapiro-Wilk para establecer asociaciones entre grupos y verificar la normalidad de la muestra, respectivamente. El nivel de significancia adoptado para las pruebas estadísticas fue del 5% (p ≤ 0,05), basado en las pruebas de Kruskal-Wallis, exacta de Fisher y *post hoc* de Dunn-Bonferroni. La confiabilidad del instrumento se midió por medio de la consistencia interna con el cálculo del coeficiente alfa de Cronbach, alcanzando un valor satisfactorio de 0,86. 

 En los resultados del CAS/CQ para la calidad de la comunicación y la colaboración entre profesionales, se adoptaron estadísticas simples. Es importante mencionar que las puntuaciones relativas al clima de seguridad pueden variar desde cero (peor percepción del clima de seguridad) hasta 100 (mejor percepción del clima de seguridad), considerándose valores mayores o iguales a 75 una percepción positiva de la seguridad del paciente ^(^ ^17^ ^)^ . Cuanto menor sea la puntuación, más frágil será la cultura de seguridad en el ambiente investigado. 

 El análisis de la información cualitativa, a partir de discusiones de la técnica GF y CF, fue guiado por un análisis de contenido temático, consistente en preanálisis, exploración del material, y tratamiento de los resultados obtenidos e interpretaciones ^(^ ^18^ ^)^ . El preanálisis corresponde a los primeros contactos del investigador con el material. Una vez en posesión del conjunto de información, éste fue organizado para dar respuesta a los objetivos, y se realizó una lectura flotante y exhaustiva para que el investigador pudiera establecer un mayor contacto con el texto. La exploración del material es la fase de codificación de datos brutos, en la que se buscó el significado central del texto separando palabras, oraciones o párrafos, que fueron clasificados y agregados en categorías. Finalmente, en el tratamiento de los resultados obtenidos e interpretaciones, los resultados brutos fueron tratados de manera significativa y válida. En este punto, por medio de resultados fieles, se analizó la información obtenida, dando interpretaciones y propósitos a los objetivos vistos anteriormente ^(^ ^18^ ^)^ . 

 Una interfaz entre los hallazgos cualitativos y cuantitativos permite un análisis más completo y profundo de los sucesos en cuestión, lo que permite encontrar patrones, obtener conocimientos y una comprensión más integral. Además, las transcripciones literales, narrativas y fotografías se organizaron en archivos utilizando el programa *NVivo 11* . 

 La integración de datos se produjo con base en el enfoque explicativo secuencial, permitiendo la conexión entre elementos cuantitativos y cualitativos, con el fin de complementarse y permitir una comprensión holística del fenómeno en estudio, y verificar si los datos cualitativos convergían o no con los cuantitativos ^(^ ^19^ ^)^ . Para la complementariedad de los datos, se exploraron las inferencias obtenidas de los GF. Esta integración fue posible gracias a una joint display, un enfoque esclarecedor para demostrar la integración CUAN→Cual que representa visualmente la integración en diseños de métodos mixtos ^(^ ^20^ ^)^ . Los datos integrados fueron aquellos referidos en los grupos focales con adhesión directa a uno o más dominios del CAS/CQ, y que guiaron la primera etapa del estudio. De esta manera, la integración se produjo de forma conectada e inductiva. 

### Aspectos éticos

El estudio cumplió con aspectos éticos y legales, destacando que todos los participantes fueron informados sobre las implicaciones de la investigación al firmar el Formulario de Consentimiento Libre e Informado, y fueron identificados con la letra P seguida del número de participación asignado, GF, fecha y categoría, para garantizar el anonimato. Se presentan fotografías para ilustrar los aspectos más destacados de la investigación. El proyecto fue aprobado por el Comité de Ética en Investigación de la institución del campo de estudio bajo dictamen número 4.092.333/2020 y Certificado de Presentación de Apreciación Ética 31032220.9.0000.5335.

## Resultados

 Los datos de la fase cuantitativa, referentes a la caracterización de los profesionales de la salud y de áreas de apoyo que actúan en los cinco agrupamientos de CQ de la institución, obtenidos por medio del CAS/CQ, se presentan en la Tabla 1 . 

**Tabla 1:** - Caracterización de los profesionales de la salud y de áreas de apoyo que trabajan en los CQ con base en el CAS/CQ ^*^ respecto de las variables género, edad, etnia, categoría profesional, experiencia profesional, tiempo de trabajo, régimen de trabajo y turno. Porto Alegre, RS, Brasil, 2021

**Variables**	**N** ^†^ **= 172**
**Género (%)** ‡	
Femenino	124 (74,7)
Masculino	42 (25,3)
**Edad (años)** §	36,8 ± 8,8
**Etnia (%)** ‡	
Blanco	126 (7,3)
Negro	18 (11,0)
Marrón	12 (7,2)
Afrodescendiente	9 (5,5)
**Categoría profesional (%)** ‡	
Instrumentador o circulante	100 (58,4)
Enfermero del Centro Quirúrgico	22 (12,8)
Cirujano/cirujano asistente	16 (9,3)
Residente de cirugía o interno	9 (5,3)
Jefe de enfermería	7 (4,0)
Administrativo	6 (3,5)
Anestesiólogo	5 (2,9)
Perfusionista	3 (1,6)
Residente de anestesia	1 (0,6)
Otros	3 (1,6)
**Experiencia profesional (años)** ||	7,0 (3,0 – 14)
**Tiempo de trabajo (años)** ||	5,0 (2,0 – 10)
**Régimen de trabajo (%)** ‡	
Tiempo parcial (36 h)	76 (47,2)
Tiempo completo (40 h)	51 (31,7)
Contratado	28 (17,4)
Cooperado	5 (3,1)
Otro	1 (0,6)
**Turno (%)** ‡	
Tiempo parcial	82 (54,7)
Tiempo completo	44 (29,3)
Variable	14 (9,3)
Nocturno	10 (6,7)

* CAS/CQ = Cuestionario de Actitudes de Seguridad/Centro Quirúrgico;

† N = Número absoluto;

§ Media ± desviación estándar;

|| Mediana (25-75 percentiles)

‡ Número absoluto (porcentaje);

La primera parte del instrumento, que aborda la Calidad de la Comunicación y Colaboración vivida entre los profesionales durante la rutina de trabajo en el CQ, demuestra que sólo la categoría de enfermería (enfermero, instrumentador y circulante quirúrgico) alcanza la puntuación mínima (≥75), reflejando una percepción positiva de la cultura de seguridad. La puntuación media de los enfermeros de CQ es de 78,9 (DE 24,6) y de los jefes de enfermería de 79,9 (DE 24,9). Entre los instrumentadores y circulantes quirúrgicos, la media se mantiene en 78,5 (DE 22,8). En el análisis global, existe diferencia significativa entre los puntajes atribuidos por estos profesionales de enfermería y otros trabajadores de la salud, como residentes e internos de cirugía (60,6; DE 29,6; p= 0,043); perfusionistas (28,7; DE 38,5; p < 0,001); y residentes e internos de anestesia (51,4; DE 32,8; p= 0,019).

La segunda parte del instrumento, que abarca los seis dominios del CAS/CQ, presenta una variación mediana de 70,0 (61,2-85,0) a 81,2 (68,7-87,5) puntos por dominio, con una mediana global de 77,6 (63,1-88,8). El CQ denominado “F” exhibió, en general, la puntuación más baja: 71,9 (61,7-85,9). También en esta localidad, los puntajes más bajos con diferencias significativas con relación a los demás fueron identificados para Percepción de la Gestión 62,5 (52,5-75,0; p=0,016), seguido de Condición de Trabajo 68,7 (54,1-87,5; p=0,001). Además, en el análisis global, se destaca que los dominios Clima de Seguridad 78,5 (64,2-85,7; p=0,047), Percepción de la Gestión 70,0 (61,2-85,0; p=0,016) y Condición de Trabajo 68,7 (54,1-87,5; p=0,001) presentan diferencias estadísticamente significativas entre los cinco CQ agrupados.

Los resultados cualitativos obtenidos por medio de la técnica de GF y de los medios fotográficos, desde la perspectiva del pensamiento ecológico y restaurativo, se agrupan en dos categorías: (1) Comprensión de la cultura de seguridad en CQ y (2) La comunicación como recurso para la construcción de una cultura de seguridad.

 En la categoría 1 se presentan las ideas aportadas por los participantes en las reuniones de los GF sobre qué es la cultura de seguridad y su importancia para los pacientes y los equipos de salud. Durante los debates, se discutió la necesidad de explicar a los pacientes la importancia del Término de Consentimiento Quirúrgico y Anestesia, así como la correcta cumplimentación del *checklist* de cirugía segura por parte de los profesionales, destacando que esta herramienta reduce errores en las cirugías y contribuye a prácticas seguras. 

 Las declaraciones de los participantes destacan que una cultura de seguridad fortalecida contribuye a un clima de trabajo favorable, como se ilustra en los extractos: *Entienden y conocen la importancia de las herramientas [checklist de cirugía segura], solo se saltan pasos por las prisas, los errores no son los únicos, hay varios pasos que no se llevan a cabo. Lo obvio no es obvio (P6, GF 1, 06/11/2020). Equipos médicos resistentes a los protocolos [cumplimiento de los términos de consentimiento quirúrgico y anestesia] (P4, GF 1, 16/11/2020).*

 La Figura 1 muestra el *checklist* de cirugía segura en la institución investigada, la cual se completa en el sistema en etapas: antes de la inducción anestésica con la participación del paciente, antes de la incisión quirúrgica y antes de que el paciente abandone el quirófano. 

**Figura 1:** - Foto autoral del uso del *checklist* de cirugía segura

 Además, en los quirófanos se coloca un panel con la información más importante a completar para cada procedimiento quirúrgico, que guía el cumplimiento de los pasos de *time out* (pausa quirúrgica) y *sign out* (antes de que el paciente abandone el quirófano), como se muestra en la Figura 2 . 

**Figura 2:** - Foto autoral del panel informativo del *checklist* colocado en los quirófanos

 Existen iniciativas por parte de la institución para valorar los principios de seguridad por medio de la implementación de paneles para el *checklist* de cirugía segura y de carteleras para difundir información institucional y sectorial. Estos aspectos se abordan en los GF y en la CF. 

 Los participantes destacan que muchos equipos médicos se niegan a aplicar los términos de consentimiento quirúrgico y anestesia, alegando falta de tiempo. Este mismo argumento se utiliza para no realizar el *checklist* de cirugía segura, aunque, según los profesionales de enfermería mencionan, este procedimiento requiera dos o tres minutos. 

La categoría 2 aborda los temas de accesibilidad de los gestores, información compartida y empatía. Cabe destacar que la comunicación entre los equipos médicos y de enfermería se desarrolla fluidamente, y que la interacción diaria entre los trabajadores facilita el proceso de comunicación.

 En los GF, los participantes confirman esta debilidad, no identificando un liderazgo presente y activo en los CQ que tienen puntajes más bajos. Por otro lado, en los debates, los profesionales de CQ con puntuaciones positivas destacan la accesibilidad al liderazgo del sector y el reconocimiento profesional como elementos importantes para el trabajo en equipo, como en las siguientes afirmaciones: *[...] algunos no tienen acceso para resolver temas, debería haber más reuniones para explicar de qué quiere hablar el grupo, eso hace falta (P4, GF 1, 06/11/2020). Estoy de acuerdo con más reuniones (P1, GF 2, 06/11/2020).*

 En las discusiones grupales también se menciona la insuficiencia de personal, vinculada a la alta rotación de la institución, debido a la falta de reconocimiento y retención de profesionales. Los empleados expresan que se sienten abrumados y agotados, sentimiento que verbalizan: *Falta de responsabilidad de los médicos, anestesiólogos, mejorar la dotación de enfermería, reducir el ausentismo (P6, GF 1, 06/11/2020). Resistencia de los médicos en los procesos (P4, GF 1, 06/11/2020). No respetan [médicos] (P1, GF 1, 06/11/2020). [...] se van por falta de equipo, de compañerismo, estamos perdiendo mucha gente buena, falta de reconocimiento, motivación. Se elogia lo malo y se desmotiva lo bueno (P4, GF 2, 06/11/2020). Las personas se sienten abrumadas, están agotadas, cansadas (P2, GF 2, 06/11/2020).*

En este apartado de informes, además de la sobrecarga y el agotamiento, se nota una percepción de poco compromiso por parte de otras categorías, como la de los médicos, lo que merece ser destacado. Además, se observa que los sentimientos relacionados con la falta de reconocimiento y motivación tienen una influencia importante en la dedicación y ejecución del trabajo. Estos últimos tienen una conexión intrínseca con el rol de liderazgo de enfermería y, normalmente, las mejoras no generan costos, por lo que sería oportuno explorar este aspecto.

 También declaran faltar reuniones con los equipos como estrategia para compartir información entre los profesionales de la salud y contribuir a la seguridad del paciente. Entre las acciones implementadas y destacadas en las CF se encuentra el uso de carteleras, cuyo objetivo es facilitar la comunicación ( Figura 3 ). Los participantes citan las carteleras en el pasillo y espacios de descanso como un recurso importante para transmitir información institucional, como resultados económico-financieros, indicadores de calidad y eventos adversos mensuales, según las declaraciones: *Creo que comunica mucho [más], es importante que [los profesionales] conozcan los objetivos (P1, GF 2, 11/02/2021). A ellos [los profesionales] les gusta saber los resultados. (P2, GF 2, 11/02/2021). Necesitamos acercarnos a los técnicos, transmitirles los objetivos, lo que logramos en el mes (P1, GF 2, 11/02/2021).*

 Los extractos de las declaraciones de los participantes que sustentan las categorías temáticas se presentan en la joint display ( Figura 4 ) de manera integrada con los datos cuantitativos, y buscan apoyar la comprensión de los resultados obtenidos por el cuestionario CAS/CQ. 

**Figura 3:** - Foto autoral de la cartelera

**Figura 4:** - Integración de los resultados cuantitativos y cualitativos mediante *joint display*

**Resultados cuantitativos**	**Resultados cualitativos**	**Integración**
Dominio 1: Clima de seguridad – en el análisis general obtiene una puntuación superior a 78,5, lo que denota una cultura de seguridad positiva. Existe una diferencia estadísticamente significativa entre los cinco CQ ^†^ agrupados (p=0,047). El CQ ^†^ F obtiene una puntuación negativa (71,4) y el CQ ^†^ G ^‡^ presenta tiene una puntuación límite de 75,0. El resto está por encima del punto de corte.	En los GF ^*^ se analizan estrategias como la reformulación del *checklist,* facilitando el llenado de información y evitando perder tiempo en elementos irrelevantes.	Se observaron avances hacia el fortalecimiento de una cultura de seguridad en las acciones descritas en los GF ^*^ y en los paseos fotográficos, destacándose mejoras en la información en las carteleras, así como la implementación de paneles con el *checklist* de cirugía segura. Estas estrategias son vistas como un recurso para difundir la cultura de seguridad del paciente en la institución y ayudan a explicar la percepción positiva obtenida por las puntuaciones en la mayoría de los CQ ^†^ en el dominio Clima de Seguridad.
Dominio 2: Percepción de la Gestión – en el análisis global, es uno de los dominios con menor puntuación (mediana 70) entre los CQ ^†^ . Los CQ ^†^ F ^‡^ , G ^‡^ y H ^‡^ presentan debilidades con puntajes inferiores a 75 puntos. Los CQ ^†^ agrupados A+B ^‡^ y C+D+E ^‡^ presentan percepciones positivas. Al comparar los grupos, se encontró que el CQ ^†^ F ^‡^ tiene la mediana más baja (62,5), con diferencia significativa (p=0,016) en relación a los CQ ^†^ A+B ^‡^ (80) y C+D+E ^‡^ (80).	Este dominio aborda la aprobación o desaprobación de acciones de liderazgo relacionadas con cuestiones de seguridad.	La Percepción de la Gestión presenta debilidades en la mayoría de los CQ ^†^ en ambas etapas. El perfil de liderazgo poco presente y activo es un aspecto destacado en los CQ ^†^ con puntuaciones más bajas. Este dominio se puede mejorar mediante conversaciones individuales, *feedbacks* y reuniones de alineación con los gestores. Este aspecto fue destacado por los participantes del GF ^*^ (grupo B) cuando mencionaron no identificar la presencia del gestor en el ambiente de cuidado.
Dominio 4: Condición de Trabajo – tiene una puntuación de 68,7 entre los CQ ^†^ . En el análisis general, es el dominio con menor puntaje, indicando mayores debilidades en la construcción de una cultura de seguridad en los CQ ^†^ evaluados. Sólo el CQ ^†^ G ^‡^ presenta puntaje favorable (83,3), con diferencia significativa (p=0,001) en relación a los CQ ^†^ A+B ^‡^ y F ^‡^ , ambos con 66,6. Los CQ ^†^ restantes tienen puntuaciones limítrofes o por debajo del punto de corte de 75.	Este dominio está relacionado con la percepción de la calidad del ambiente de trabajo por parte de los profesionales. En los GF ^*^ se señala la sobrecarga de trabajo, ligada a la intensa dinámica en el día a día del CQ ^†^ . Además, se destaca la importancia de la asistencia entre profesionales.	La Condición de Trabajo presenta una percepción negativa entre los CQ ^†^ en la etapa cuantitativa, hecho reforzado en las declaraciones de los participantes. La falta de personal y la intensa dinámica de trabajo sobrecargan al trabajador. Hay oportunidades de mejora cambiando el tamaño de los equipos, fomentando la colaboración entre pares y revisando los cronogramas de actividades diarias.
Dominio 5: Comunicación en el Ambiente Quirúrgico – tiene mejores puntuaciones en general y en todos los CQ ^†^ , y demuestra una percepción positiva sobre la calidad de la comunicación y colaboración en el equipo multidisciplinario. En la puntuación global tiene una mediana de 81,2. Sólo el CQ ^†^ F ^‡^ presentó un puntaje límite, 75,0, sin diferencia significativa entre los grupos (p=0,101).	Los participantes destacan la importancia de la interacción entre los equipos de salud como fundamental en el trabajo diario, junto con la comunicación.	A pesar del puntaje positivo en la etapa cuantitativa, en la etapa cualitativa se destaca la necesidad de un mayor número de reuniones e interacción entre los equipos de salud. Si bien se identifica que el uso de *WhatsApp* contribuye a la agilidad de la comunicación entre equipos, los espacios de intercambio y diálogo pueden mejorar la comunicación y fortalecer la cultura de seguridad.

* GF = Grupo focal;

‡ Letras de designación de cada uno de los centros quirúrgicos investigados

† CQ = Centro Quirúrgico;

 Los dominios 3 (Percepción del Estrés) y 6 (Percepción del Desempeño Profesional) en la etapa cuantitativa presentan puntuaciones positivas. El dominio 3 tiene la puntuación más alta en la evaluación global (81,2), mientras que el dominio 6 tiene una mediana de 75,0 en el análisis global. Los CQ A+B y G tienen mejores puntuaciones (81,2). El resto tiene puntuaciones límite. Como estos temas no se discutieron en los GF, estos dominios no se incluyeron en la *joint display* para la integración de los datos. 

## Discusión

 Los datos que caracterizan la muestra del presente estudio son consistentes con otros hallazgos en el contexto brasileño, indicando un predominio de mujeres (74,7%), profesionales de enfermería 126 (75,2%), con una mediana de edad de 37 años, trabajando en los CQ. Una investigación realizada en Brasil en un CQ de un hospital universitario, con el objetivo de evaluar el riesgo de enfermedades patógenas en estos trabajadores, mostró que la mayoría de los integrantes de este sector son mujeres (81,9%) con edades entre 36 y 50 años, corroborando los hallazgos de este estudio ^(^ ^21^ ^)^ . 

 La primera parte del instrumento CAS/CQ demostró que sólo la categoría de enfermería (enfermero, instrumentador y circulante quirúrgico) alcanzó la puntuación mínima (≥75) para ser considerada una percepción positiva de la cultura de seguridad. En otro estudio ^(^ ^22^ ^)^ , los enfermeros también obtuvieron puntuaciones medias más altas en ítems relacionados con la calidad de la comunicación y colaboración, similar a los resultados de este estudio. Un estudio realizado en el CQ de un hospital público universitario del estado de Paraná, que encontró una puntuación más baja entre los instrumentadores/circulantes, sugiere que existe una baja calidad de comunicación y colaboración en las otras categorías que actúan en el CQ ^(^ ^21^ ^)^ . 

 La comunicación y colaboración adecuadas entre los miembros del equipo del CQ son fundamentales para garantizar la seguridad del paciente durante la cirugía, por lo que todos tienen un papel importante que desempeñar, y es esencial que cada uno comprenda cada función y trabaje en conjunto ^(^ ^22^ ^)^ . 

 La comunicación efectiva entre los profesionales de la salud, los pacientes y las familias es un factor crucial para brindar un cuidado seguro y de calidad. Es la base para la toma de decisiones compartida, la identificación temprana de problemas y la resolución de conflictos, todos elementos fundamentales del cuidado centrado en el paciente. Desde esta perspectiva, el enfermero juega un papel destacado en la integración de la información entre equipos, reduciendo las posibilidades de que ocurran eventos adversos y promoviendo una comunicación abierta y honesta entre los miembros de los equipos ^(^ ^23^ ^-^ ^24^ ^)^ . 

 Cuando se establece una cultura de seguridad en una institución, la comunicación entre profesionales se vuelve más fácil y efectiva. Esto se debe a que existe una mayor confianza entre los miembros del equipo, quienes se sienten más cómodos comunicándose abiertamente y compartiendo información relevante. Además, cuando todos los miembros del equipo conocen las medidas de seguridad adoptadas y comprenden la importancia de seguirlas, se mejora el trabajo en equipo y la colaboración, lo que contribuye a brindar un cuidado de calidad al paciente ^(^ ^25^ ^)^ . 

El análisis descriptivo general de la puntuación por agrupación de CQ, con base en los dominios del CAS/CQ, reveló una percepción favorable del clima de seguridad en los lugares considerados. Esto indica una percepción positiva sobre la seguridad del paciente en estos ambientes. Entre los dominios del CAS/CQ, Percepción de la Gestión y Condición de Trabajo se destacaron como los más débiles en el CQ evaluado. Esto sugiere que los empleados de estos centros pueden tener una percepción menos positiva sobre la gestión y las condiciones laborales existentes. Puede ser necesario mejorar estos aspectos para promover una cultura de seguridad más sólida.

Por otro lado, los dominios Comunicación en el Ambiente Quirúrgico, Clima de Seguridad, Percepción del Desempeño Profesional y Percepción del Estrés presentaron puntuaciones favorables. Esto indica que los empleados de los CQ tienen una percepción positiva en estos aspectos, lo que sugiere la existencia de una cultura de seguridad más sólida y una buena comunicación entre los profesionales involucrados.

 El análisis integrado de los resultados refuerza la fragilidad de los dominios Percepción de la Gestión y Condición de Trabajo en los CQ evaluados, tanto por las puntuaciones negativas como por las declaraciones de los profesionales en los grupos focales. Es interesante señalar que también se encontraron resultados similares en otros estudios sobre las percepciones gerenciales y las condiciones laborales en los CQ. Esta consistencia en los resultados sugiere que la baja percepción de la gestión, sumada a las condiciones laborales desfavorables, puede llevar a los profesionales de la salud a desconocer el compromiso de la gestión de las instituciones con los factores ocultos de la cultura de seguridad. En consecuencia, este desconocimiento puede reflejarse en puntuaciones negativas e indicar un distanciamiento entre el equipo y sus superiores ^(^ ^26^ ^-^ ^27^ ^)^ . 

 Para promover el desarrollo de una cultura de seguridad del paciente, es necesario invertir y desarrollar capacitaciones e involucrar a los profesionales de la salud en este tema. Además, es fundamental que los hospitales proporcionen los recursos de apoyo necesarios a los profesionales de la salud ^(^ ^28^ ^)^ . 

El puntaje positivo en el dominio Clima de Seguridad fue reforzado por las declaraciones de los participantes en la etapa cualitativa, destacando la importancia de una cultura de seguridad fortalecida para crear un clima de trabajo favorable.

 Es interesante señalar que un estudio realizado en tres hospitales públicos y privados del estado de Rio Grande do Sul comparó el clima de seguridad de esas instituciones, e identificó puntuaciones positivas en el servicio privado, mientras que las instituciones filantrópicas presentaron puntuaciones inferiores a la media, enfatizando una debilidad en la cultura de seguridad en estas instituciones ^(^ ^29^ ^)^ . 

Estos hallazgos resaltan la importancia de una cultura de seguridad sólida para promover un clima de trabajo positivo y seguro en los CQ. Una cultura de seguridad establecida es esencial para fomentar la comunicación abierta, el aprendizaje continuo y el compromiso de todos los profesionales de la salud en la identificación y prevención de eventos adversos.

 El dominio Percepción del Estrés también presentó una puntuación superior a la media en otro estudio, lo que indica que los participantes perciben factores estresantes en el ambiente laboral ^(^ ^30^ ^)^ . Esta percepción puede ser especialmente relevante para los profesionales que trabajan en CQ, ya que están expuestos a una serie de tensiones debido al entorno laboral desafiante. 

 Los profesionales que trabajan en CQ enfrentan altos niveles de estrés debido a varios factores, como ambiente cerrado, riesgos involucrados, diferentes rutinas, necesidad de habilidades técnicas y alta productividad requerida ^(^ ^31^ ^)^ . Estos elementos pueden contribuir a la presión emocional y psicológica que enfrentan los profesionales quirúrgicos. 

 Ante este escenario, se sugiere reorganizar actividades y redimensionar equipos como medidas para mejorar la calidad de vida en el trabajo y reforzar la seguridad del paciente en el ambiente quirúrgico ^(^ ^26^ ^)^ . Reevaluando la distribución de tareas y carga de trabajo, es posible promover un equilibrio más saludable para los profesionales, reduciendo el estrés y aumentando el bienestar en el trabajo. 

La puntuación límite en el dominio Percepción del Desempeño Profesional indica una evaluación neutral por parte del equipo quirúrgico en este aspecto, según los resultados del CAS/CQ. Este dominio está relacionado con la forma en que la fatiga y la sobrecarga laboral afectan el desempeño profesional de los individuos.

 El desempeño profesional está intrínsecamente ligado a la satisfacción laboral, reflejando la experiencia individual de cada profesional. La satisfacción laboral se define como un sentimiento positivo respecto al trabajo, que abarca aspectos como el contenido del trabajo, las oportunidades de desarrollo, el reconocimiento, las condiciones laborales y las relaciones con compañeros y superiores ^(^ ^30^ ^)^ . 

Es importante considerar que la satisfacción laboral y el desempeño profesional se ven afectados por una variedad de factores, incluidas las condiciones laborales, el apoyo organizacional, el reconocimiento y las oportunidades de crecimiento. Por ello, es fundamental que las instituciones de salud sean conscientes de estos aspectos y adopten medidas para promover un ambiente de trabajo saludable y equilibrado, que valore el bienestar de los profesionales.

 La satisfacción laboral no sólo influye en el bienestar y la salud de los profesionales, sino que también está relacionada con la prevención de enfermedades profesionales y la obtención de mejores resultados en el proceso de trabajo, que incluye las actividades que realizan las enfermeras y otros miembros del equipo de salud ^(^ ^31^ ^)^ . 

Cuando los profesionales están satisfechos en el trabajo, tienden a estar más comprometidos, motivados y dedicados a sus responsabilidades. Esto puede conducir a una mayor eficiencia, productividad y calidad de la atención al paciente. Por otro lado, la insatisfacción laboral puede derivar en un ambiente negativo, desmotivación, aumento del estrés y posibles errores o fallas en la prestación de cuidados.

Como limitaciones del estudio se destaca el reducido número de participantes en algunas categorías profesionales, debido al período crítico de salud provocado por la pandemia de Covid-19 y la reducción significativa de los horarios quirúrgicos. La ausencia de representantes en el GF de todas las categorías investigadas en la primera etapa, incluidos médicos, perfusionistas y auxiliares de farmacia o higiene, es una limitación. Sin embargo, el estudio ofrece reflexiones relevantes sobre el clima y las actitudes de seguridad en el CQ, y puede ser considerado como una orientación asertiva para un cuidado seguro.

Los resultados del estudio indican una mejor percepción del clima de seguridad por parte del equipo de enfermería, lo que señala un mayor potencial para su compromiso en la construcción de una cultura de seguridad. En este sentido, destaca la necesidad de una evaluación más profunda de las estrategias de involucramiento de los equipos médicos, con el objetivo de promover un trabajo interprofesional y colaborativo.

Las contribuciones de este estudio a la práctica asistencial incluyen resaltar la importancia de formar profesionales que ocupen puestos directivos, buscando una mayor aproximación y comprensión de las necesidades del equipo asistencial. Esto implica un enfoque más asertivo y sensible para promover un ambiente de trabajo saludable y seguro.

Además, el estudio destaca la necesidad de invertir en las condiciones laborales que se ofrecen a los empleados. Esto implica ajustar la carga de trabajo, considerar una distribución equitativa de las tareas y revisar la dotación de personal. Al proporcionar una carga de trabajo adecuada, los centros quirúrgicos pueden promover un clima de seguridad más positivo, reduciendo el riesgo de errores y mejorando la calidad del cuidado brindado. Estas sugerencias tienen como objetivo mejorar la calidad de la atención ofrecida, promoviendo un ambiente de trabajo más colaborativo, seguro y eficaz para todo el equipo asistencial.

## Conclusión

Los CQ estudiados presentaron resultados que indican una percepción positiva del clima de seguridad entre los profesionales de salud y de apoyo. Los dominios Percepción del Estrés, Comunicación en el Ambiente Quirúrgico, Clima de Seguridad y Percepción del Desempeño Profesional obtuvieron puntuaciones más altas en relación a la cultura de seguridad, siendo esto reforzado en el análisis cualitativo.

El análisis realizado en esta investigación sobre las actitudes en materia de seguridad en los CQ proporcionó una comprensión profunda de los desafíos que enfrenta este complejo ambiente. La combinación de datos cuantitativos y cualitativos, que es un enfoque aún poco explorado en la enfermería perioperatoria, resultó ser un desafío, pero fructífero.

Si bien el análisis global del dominio de Comunicación y Colaboración entre Equipos fue positivo, se identificaron oportunidades de mejora en este proceso tanto en datos cuantitativos como cualitativos. El equipo de enfermería parece adherirse con mayor facilidad a los procesos y rutinas institucionales orientados a la seguridad del paciente, lo que refleja un mayor fortalecimiento cultural de esta categoría en relación al cuidado seguro.

# Translation: Desafios da cultura de segurança em Centro Cirúrgico: estudo de métodos mistos ^*^

**DOI:** 10.1590/1518-8345.7001.4207

**Authors:** Nery José de Oliveira Junior, Caren de Oliveira Riboldi, Daniela Campos de Andrade Lourenção, Vanessa de Brito Poveda, João Lucas Campos de Oliveira, Ana Maria Müller de Magalhães

**Affiliations:** 1 Universidade Federal do Rio Grande do Sul, Escola de Enfermagem, Porto Alegre, RS, Brasil.; 2 Universidade de São Paulo, Escola de Enfermagem, São Paulo, SP, Brasil.

**Keywords:** Segurança do Paciente, Cultura Organizacional, Enfermagem Perioperatória, Centros Cirúrgicos, Enfermagem, Ambiente de Instituições de Saúde

## Abstract

**Objetivo:**

analisar as atitudes de segurança de profissionais da saúde e de áreas de apoio atuantes em Centro Cirúrgico.

**Método:**

estudo explanatório sequencial de métodos mistos. A etapa quantitativa abrangeu 172 profissionais da saúde e de apoio em oito Centros Cirúrgicos de um complexo hospitalar. Aplicou-se o Questionário de Atitudes de Segurança/Centro Cirúrgico *.* Na etapa qualitativa subsequente, 16 profissionais participaram do Grupo Focal. Utilizaram-se métodos fotográficos na perspectiva do pensamento ecológico e restaurativo, e a análise dos dados ocorreu de maneira integrada, por conexão.

**Resultados:**

o escore geral, por agrupamento de Centros Cirúrgicos, com base nos domínios do Questionário de Atitudes de Segurança/Centro Cirúrgico, revela percepção favorável do clima de segurança, com destaque para os domínios Percepção do Estresse, Comunicação no Ambiente Cirúrgico, Clima de Segurança e Percepção do Desempenho Profissional. A análise global do domínio Comunicação e Colaboração entre as Equipes mostra-se positiva e é corroborada pelos dados da etapa qualitativa, na qual destaca-se a importância da interação e da comunicação entre as equipes de saúde como fundamentais para o trabalho diário.

**Conclusão:**

a percepção das atitudes de segurança entre os profissionais da saúde e de apoio foi positiva. A percepção da equipe de enfermagem se destaca como mais próxima ou favorável a atitudes condizentes com a cultura de segurança.

## Destaques

**(1)** A comunicação e o trabalho em equipe reforçam a cultura de segurança.

**(2)** Equipe de enfermagem mais engajada para a cultura de segurança.

**(3)** Percepção favorável do clima de segurança pelos profissionais de saúde e de apoio.

**(4)** Adesão da equipe de enfermagem aos processos e rotinas institucionais.

## Introdução

 A Organização Mundial da Saúde destaca que todo ano, em países de baixa e média renda, ocorrem aproximadamente 134 milhões de eventos adversos e 2,6 milhões de mortes associadas a ambientes inseguros, atestando que a segurança do paciente deve ser uma meta permanente. Neste contexto, embora existam avanços nas evidências sobre o impacto dos eventos adversos, ainda são incipientes as medidas de redução, mitigação, prevenção e mensuração dos riscos potenciais em termos de tecnologia, recursos financeiros e intervenções ^(^ ^1^ ^)^ . Para que melhorias sejam instituídas, faz-se necessário fomentar uma cultura que favoreça a segurança do paciente nas organizações de saúde ^(^ ^2^ ^)^ . 

 A cultura de segurança é compreendida como um conjunto de valores e práticas individuais e coletivas com foco na redução de riscos e danos ao paciente ^(^ ^3^ ^)^ . Como se trata de um elemento cultural e eminentemente multifacetado, que emana de uma concepção organizacional, existem fatores que são considerados mensuráveis ^(^ ^4^ ^)^ . Um exemplo é o clima de segurança, referente à percepção dos atores organizacionais sobre as políticas e práticas (in)seguras no ambiente de cuidados em saúde ^(^ ^3^ ^-^ ^4^ ^)^ . 

 Os meios de análise da cultura e do clima de segurança incluem questionários, entrevistas e observações, ou ainda, a avaliação externa por especialistas no assunto. Tais análises permitem verificar o nível do comprometimento institucional e dos profissionais com valores, crenças, recursos, atitudes e comportamentos relacionados com a segurança do paciente ^(^ ^1^ ^,^ ^4^ ^-^ ^5^ ^)^ . Em outras palavras, ainda que na prática afira-se o clima de segurança por meio da dinâmica laboral, incluindo principalmente a percepção dos trabalhadores, isso será um reflexo de como a cultura organizacional posiciona-se com relação ao cuidado seguro ^(^ ^2^ ^,^ ^5^ ^)^ . 

 As pesquisas relacionadas com a cultura de segurança no ambiente hospitalar estão em constante crescimento ^(^ ^6^ ^-^ ^7^ ^)^ , o que reforça o fato de que os valores organizacionais impactam diretamente os resultados de segurança, abarcando também a ocorrência de eventos adversos, ou seja, danos concretos ao paciente ^(^ ^8^ ^)^ . Tais valores precisam se fortalecer em qualquer ambiente de prestação assistencial, ainda que se reconheça que alguns ambientes possuem risco mais elevado ou evidente pela natureza dos processos de trabalho desenvolvidos, a exemplo do Centro Cirúrgico (CC) ^(^ ^9^ ^)^ . 

 Os chamados *near misses* ou *quase erros* já foram menos frequentes nos CC do que em outras unidades, como na internação, terapia intensiva e pediatria ^(^ ^10^ ^)^ . Em contrapartida, a ocorrência de eventos adversos de altíssima severidade — conhecidos como *never events* — é alarmante quando trata-se do período intraoperatório e pós-operatório imediato, como cirurgia em lateralidade errada, procedimento e/ou paciente errado, retenção de material no paciente e queimadura por eletrocautério ^(^ ^11^ ^)^ . Soma-se a isso o fato de que até 90% dos eventos adversos relacionados com o ato cirúrgico são classificados como preveníveis ^(^ ^12^ ^)^ , tornando imperativo que as medidas de segurança do paciente no CC sejam instituídas e estimuladas, o que pode ser realizado com maior assertividade por meio de análises sistemáticas das atitudes de segurança nesses ambientes. 

Ante o exposto, esta pesquisa fundamentou-se nas seguintes questões norteadoras: Qual é a percepção do clima de segurança entre os profissionais da saúde e de áreas de apoio atuantes em CC? Quais os aspectos que repercutem na cultura de segurança nesse cenário? Logo, o objetivo deste trabalho consistiu em analisar as atitudes de segurança entre profissionais da saúde e de áreas de apoio atuantes em CC.

## Método

### Delineamento do estudo

 Trata-se de um estudo de métodos mistos, ancorado no desenho explanatório sequencial, que combina elementos quantitativos e qualitativos para responder às questões de pesquisa de forma mais abrangente e completa. A primeira fase, de abordagem quantitativa e maior peso (QUAN), permitiu mensurar variáveis específicas de maneira objetiva, enquanto a etapa posterior, de abordagem qualitativa secundária (Qual), visou aprofundar a compreensão do fenômeno previamente aferido. A análise inicial dos resultados quantitativos orientou a coleta dos dados qualitativos (QUAN→Qual), subsidiando *insights* a serem explorados em maior detalhe e facilitando a descoberta de novos sentidos, interpretações e relações entre as variáveis ^(^ ^13^ ^)^ . 

### Cenário da coleta de dados

O estudo foi conduzido em um único complexo composto por oito hospitais, situado na cidade de Porto Alegre-RS, Brasil. Cada local dispõe de um CC, totalizando 53 salas destinadas a cirurgias gerais e de especialidades. Ao todo, a produtividade cirúrgica é de aproximadamente 6.000 procedimentos por mês. Os CC contemplados nesta pesquisa caracterizam-se da seguinte maneira: CC “A” (13 salas), que realiza cirurgias de diversas especialidades; CC “B” (4 salas), destinado a procedimentos oftalmológicos; CC “C” (3 salas), que realiza cirurgias da especialidade de pneumologia; CC “D” (3 salas), que realiza cirurgias neurológicas; CC “E” (4 salas), que realiza cirurgias cardíacas; CC “F” (7 salas), que atende a especialidade de oncologia; CC “G” (12 salas), que contempla as especialidades da cirurgia plástica e transplantes; e CC “H”(7 salas), que atende a especialidade de pediatria.

### Período

O estudo transcorreu entre junho de 2020 e fevereiro de 2021.

### População

A população do estudo foi constituída pelos profissionais de saúde e de áreas de apoio dos oito CC. Os profissionais de saúde foram: médicos cirurgiões e anestesiologistas, residentes de medicina, enfermeiras e técnicos de enfermagem. Assistentes administrativos, auxiliares de farmácia e de higienização foram considerados como áreas de apoio.

### Critérios de seleção

Os critérios de elegibilidade para a participação no estudo consistiram em compor a equipe de um dos CC e não estar em férias ou afastamento funcional no decorrer da coleta de dados.

### Participantes

A etapa quantitativa abrangeu 172 profissionais, distribuídos entre as seguintes categorias: instrumentador cirúrgico e circulante (100), enfermeiro de CC (22), cirurgião ou cirurgião assistente (16), residente de cirurgia ou interno (9), enfermeiro-chefe (7), assistente administrativo (6), anestesiologista (5), perfusionista (3), residente de anestesiologia (1) e auxiliares de farmácia ou higienização (3). Ressalta-se que o momento era de baixa realização de cirurgias devido às restrições do período pandêmico de Covid-19, que repercutiram em menor presença das equipes médicas, uma vez que as agendas cirúrgicas foram reduzidas. Estas restrições impactaram também a etapa subsequente, qualitativa, que contou com 16 participantes entre: enfermeiros (5), técnicos de enfermagem (10) e assistente administrativo (1). Para esta fase, além da participação na etapa anterior, era necessário atuar nos CC que apresentaram os escores mais favoráveis ou desfavoráveis na etapa quantitativa. Do primeiro encontro participaram oito representantes de cada CC, e do segundo, 12 trabalhadores, sendo seis de cada agrupamento.

### Instrumentos utilizados para a coleta das informações

Na etapa quantitativa utilizou-se o Questionário de Atitudes de Segurança/Centro Cirúrgico (SAQ/CC) com a finalidade de mensurar o clima de segurança nos serviços de saúde a partir da percepção dos profissionais acerca da segurança do paciente. Os pesquisadores optaram por uma versão menos atualizada do instrumento, pois culturalmente, no local de realização do estudo, os técnicos de enfermagem realizam a função de circulante ou instrumentador alternadamente na escala de trabalho. Na versão atual do instrumento, tais funções foram agrupadas, não se aplicando à realidade em questão.

 A versão brasileira do SAQ/CC consiste em uma escala do tipo Likert e se divide em três partes. A primeira, com 15 assertivas, é referente à qualidade da comunicação e colaboração entre os profissionais que atuam no ambiente cirúrgico, que o participante da pesquisa deve responder sobre a sua relação com cada uma das categorias profissionais. A segunda é composta por 40 assertivas, divididas conceitualmente em seis domínios: clima de segurança (sete itens), percepção da gerência (cinco itens), percepção do estresse (quatro itens), condição do trabalho (seis itens), comunicação no ambiente cirúrgico (quatro itens) e percepção do desempenho profissional (quatro itens). A terceira parte abrange informações demográficas (sexo, raça/etnia, categoria profissional, tempo de atuação, turno de atuação, entre outros) e um espaço onde o participante pode escrever três recomendações para a melhoria da segurança do paciente em CC, e indicar se já havia respondido o instrumento anteriormente ^(^ ^14^ ^)^ . 

 A etapa qualitativa posterior utilizou a técnica de Grupo Focal (GF) e os métodos fotográficos de pesquisa na perspectiva do pensamento ecológico e restaurativo, por meio de caminhada fotográfica (CF) ^(^ ^15^ ^-^ ^16^ ^)^ . Nessa etapa, a amostra foi intencional e por conveniência, entre aqueles que participaram da primeira etapa (quantitativa). 

Para a condução dos GF, foi utilizado um roteiro elaborado pelos pesquisadores, abordando os tópicos: cultura de segurança, o que influencia a cultura de segurança, relação da equipe multiprofissional no ambiente de CC, e clima de segurança no CC.

 A técnica de GF foi organizada e conduzida conforme o seguinte roteiro: abertura da sessão, integração dos participantes, explicação sobre a dinâmica de discussões, *setting* grupal, debate, síntese dos momentos anteriores e encerramento do encontro. Ainda, firmou-se um acordo sobre confidencialidade, reforçando que os debates e as ideias discutidas nos encontros ficariam restritos aos integrantes do grupo. 

O roteiro foi planejado a partir da necessidade de complementar/aprofundar os achados quantitativos. Portanto, a investigação destes elementos foi importante para constituir um banco de dados qualitativos, criando um repositório de informações ricas e contextuais que podem ser comprovadas, para encontrar inferências e esclarecimentos sobre os achados quantitativos.

### Coleta de dados

Na primeira fase do estudo, a coleta de dados quantitativos, foi aplicado o questionário de forma impressa e presencial, durante a jornada de trabalho dos profissionais. Seguiu-se a seleção amostral por conveniência, no período de junho a julho de 2020. A segunda fase da pesquisa, de cunho qualitativo, ocorreu entre outubro de 2020 e fevereiro de 2021. A amostra dessa etapa foi intencional e por conveniência, selecionada entre aqueles que participaram da primeira etapa (quantitativa).

Foi disponibilizada uma lista para aqueles que tivessem interesse em participar do GF e da CF nos CC, definidos de acordo com os melhores e piores resultados no questionário SAQ/CC. Os 12 primeiros inscritos para cada GF foram selecionados, e realizaram-se quatro encontros, sendo dois com os profissionais do CC com o melhor escore (grupo A) e dois com aqueles vinculados ao local com pior escore (grupo B).

O primeiro encontro de GF foi para a discussão de temas de interesse, relacionados com os aspectos da cultura de segurança e com o levantamento de tópicos considerados prioritários pelos participantes, a fim de compor o roteiro da CF. Após o primeiro GF, os tópicos abordados foram validados por um membro de cada grupo (enfermeira), que, posteriormente, acompanhou a CF pelo CC. O segundo encontro teve como foco principal a discussão das fotos obtidas, caracterizando-se como GF para elicitação fotográfica.

### Tratamento e análise dos dados

 Para fins de análise, os CC foram dispostos em cinco grupos (A+B; C+D+E; F; G; H), segundo o total de respondentes, semelhança de processos, número de salas cirúrgicas e volume de procedimentos. Os dados quantitativos oriundos da primeira etapa foram analisados no *software Statistical Package for the Social Sciences* versão 21.0. Utilizou-se estatística descritiva na caracterização da amostra, pela qual as variáveis discretas foram apresentadas na forma de média e desvio padrão ou mediana e percentil, e as variáveis categóricas expressas na forma de frequência absoluta e relativa. 

 Para a análise estatística, considerou-se como variáveis dependentes os escores dos domínios do SAQ/CC: Clima de Segurança, Percepção da Gerência, Percepção do Estresse, Condição de Trabalho, Comunicação no Ambiente Cirúrgico e Percepção do Desempenho Profissional; e como variáveis independentes: idade, sexo, profissão e tempo de experiência na especialidade. Os testes qui-quadrado e Shapiro-Wilk foram utilizados para a associação entre os grupos e verificação de normalidade da amostra, respectivamente. O nível de significância adotado para os testes estatísticos foi de 5% ( *p* ≤ 0,05), a partir dos testes de Kruskal-Wallis, Exato de Fisher e *post hoc* de Dunn-Bonferroni. A confiabilidade do instrumento foi aferida por meio da consistência interna com o cálculo do coeficiente alfa de Cronbach, atingindo valor satisfatório de 0,86. 

 Nos resultados do SAQ/CC para a qualidade da comunicação e colaboração entre profissionais, adotou-se estatística simples. É importante mencionar que os escores referentes ao clima de segurança podem variar de zero (pior percepção do clima de segurança) a 100 (melhor percepção do clima de segurança), sendo que valores maiores ou iguais a 75 são considerados uma percepção positiva da segurança do paciente ^(^ ^17^ ^)^ . Quanto menor a pontuação, mais frágil a cultura de segurança no ambiente pesquisado. 

 A análise das informações qualitativas, a partir das discussões da técnica de GF e CF, foi norteada por uma análise de conteúdo do tipo temática, composta por pré-análise, exploração do material, e tratamento dos resultados obtidos e interpretações ^(^ ^18^ ^)^ . A pré-análise corresponde aos primeiros contatos do pesquisador com o material. De posse do conjunto de informações, o mesmo foi organizado de forma a responder aos objetivos, e foi realizada uma leitura flutuante e exaustiva para que o pesquisador estabelecesse um contato maior com o texto. A exploração do material é a fase de codificação de dados brutos, na qual buscou-se o núcleo de sentido do texto através da separação de palavras, frases ou parágrafos, que foram classificados e agregados em categorias. Por fim, no tratamento dos resultados obtidos e interpretações, os resultados brutos foram tratados de maneira significativa e válida. Neste momento, por meio dos resultados fiéis, foram analisadas as informações obtidas, dando-se interpretações e propósitos aos objetivos vistos anteriormente ^(^ ^18^ ^)^ . 

 Uma interface entre os achados qualitativos e quantitativos permite uma análise mais completa e aprofundada das ocorrências em questão, possibilitando encontrar padrões, obter *insights* e uma compreensão mais abrangente. Além disso, as transcrições literais, narrativas e fotos foram organizadas em arquivos utilizando o programa *NVivo 11* . 

 A integração dos dados ocorreu a partir da abordagem explanatória sequencial, permitindo a conexão entre elementos quantitativos e qualitativos, a fim de se complementarem e permitirem uma compreensão holística do fenômeno em estudo, e verificar se os dados qualitativos convergiam ou não com os quantitativos ^(^ ^19^ ^)^ . Para a complementaridade dos dados, foram exploradas as inferências obtidas a partir dos GF. Essa integração foi viabilizada a partir de uma *joint display* , uma abordagem esclarecedora para demonstrar a integração QUAN→Qual que representa visualmente a integração nos desenhos de métodos mistos ^(^ ^20^ ^)^ . Os dados integrados foram aqueles referidos nos grupos focais com aderência direta a um ou mais domínios do SAQ/CC, e que nortearam a primeira etapa do estudo. Desta forma, a integração ocorreu de maneira conectada e indutiva. 

### Aspectos éticos

O estudo atendeu aos aspectos éticos e legais, ressaltando-se que todos os participantes foram informados sobre as implicações da pesquisa na ocasião da assinatura do Termo de Consentimento Livre e Esclarecido, e foram identificados com a letra P seguida do número de participação atribuído, GF, data e categoria, a fim de garantir o anonimato. As fotografias são apresentadas para ilustrar pontos de destaque da pesquisa. O projeto foi aprovado pelo Comitê de Ética em Pesquisa da instituição campo de estudo sob parecer de número 4.092.333/2020 e Certificado de Apresentação de Apreciação Ética 31032220.9.0000.5335.

## Resultados

 Os dados da fase quantitativa, referentes à caracterização dos profissionais de saúde e de áreas de apoio que atuam nos cinco agrupamentos dos CC da instituição, obtidos por meio do SAQ/CC, estão apresentados na Tabela 1 . 

**Tabela 1:** - Caracterização dos profissionais de saúde e trabalhadores de apoio atuantes nos Centros Cirúrgicos a partir do SAQ/CC ^*^ quanto às variáveis sexo, idade, etnia, categoria profissional, experiência profissional, tempo de trabalho, regime de trabalho e turno. Porto Alegre, RS, Brasil, 2021

**Variáveis**	**N** ^†^ **= 172**
**Sexo (%)** ^‡^	
Feminino	124 (74,7)
Masculino	42 (25,3)
**Idade (anos)** ^§^	36,8 ± 8,8
**Etnia (%)** ^‡^	
Branco	126 (7,3)
Negro	18 (11,0)
Pardo	12 (7,2)
Afrodescendente	9 (5,5)
**Categoria profissional (%)** ^‡^	
Instrumentador ou circulante	100 (58,4)
Enfermeiro de Centro Cirúrgico	22 (12,8)
Cirurgião/cirurgião assistente	16 (9,3)
Residente de cirurgia ou interno	9 (5,3)
Enfermeiro chefe	7 (4,0)
Administrativo	6 (3,5)
Anestesiologista	5 (2,9)
Perfusionista	3 (1,6)
Residente de anestesia	1 (0,6)
Outros	3 (1,6)
**Experiência profissional (anos)** ^||^	7,0 (3,0 – 14)
**Tempo de trabalho (anos)** ^||^	5,0 (2,0 – 10)
**Regime de trabalho (%)** ^‡^	
Período parcial (36 h)	76 (47,2)
Período integral (40 h)	51 (31,7)
Contratado	28 (17,4)
Cooperado	5 (3,1)
Outro	1 (0,6)
**Turno (%)** ^‡^	
Período parcial	82 (54,7)
Período integral	44 (29,3)
Variável	14 (9,3)
Noturno	10 (6,7)

* SAQ/CC = Questionário de Atitudes de Segurança/Centro Cirúrgico;

† N = Número absoluto;

§ Média ± desvio padrão;

|| Mediana (percentis 25-75)

‡ Número absoluto (porcentagem);

A primeira parte do instrumento, que aborda a Qualidade da Comunicação e Colaboração vivenciada entre os profissionais durante a rotina de trabalho no CC, demonstra que apenas a categoria de enfermagem (enfermeiro, instrumentador e circulante cirúrgico) atinge o escore mínimo (≥75), refletindo uma percepção positiva de cultura de segurança. A pontuação média dos enfermeiros de CC é de 78,9 (DP 24,6), e dos enfermeiros chefes, 79,9 (DP 24,9). Já entre os instrumentadores e circulantes cirúrgicos, a média permanece em 78,5 (DP 22,8). Na análise global, verifica-se diferença significativa entre os escores atribuídos por esses profissionais de enfermagem e demais trabalhadores da saúde, como residentes e internos de cirurgia (60,6; DP 29,6; p= 0.043); perfusionistas (28,7; DP 38,5; p < 0.001); e residentes e internos de anestesia (51,4; DP 32,8; p= 0.019).

A segunda parte do instrumento, que engloba os seis domínios do SAQ/CC, apresenta uma variação de mediana de 70,0 (61,2-85,0) a 81,2 (68,7-87,5) pontos por domínio, com mediana geral de 77,6 (63,1-88,8). O CC nomeado como “F” exibiu, de maneira geral, o menor escore: 71,9 (61,7-85,9). Também neste local foram identificados os escores mais baixos e com diferenças significativas com relação aos demais para Percepção da Gerência 62,5 (52,5-75,0; p=0,016), seguido de Condição de Trabalho 68,7 (54,1-87,5; p=0,001). Ainda, na análise global, chama a atenção que os domínios Clima de Segurança 78,5 (64,2-85,7; p=0,047), Percepção da Gerência 70,0 (61,2-85,0; p=0,016) e Condição de Trabalho 68,7 (54,1-87,5; p=0,001) apresentam diferenças estatisticamente significativas entre os cinco CC agrupados.

Os resultados qualitativos obtidos por meio da técnica de GF e dos meios fotográficos, na perspectiva do pensamento ecológico e restaurativo, estão agrupados em duas categorias: (1) Compreendendo a cultura de segurança em CC e (2) Comunicação como recurso para a construção de uma cultura de segurança.

 Na categoria 1, são apresentadas as ideias trazidas pelos participantes dos encontros dos GF sobre o que é cultura de segurança e sua importância para os pacientes e equipes de saúde. Durante os debates, discutiu-se a necessidade de explicar aos pacientes a importância do Termo de Consentimento Cirúrgico e Anestésico, bem como o preenchimento correto do *checklist* da cirurgia segura pelos profissionais, destacando que essa ferramenta diminui erros em cirurgias e contribui para práticas seguras. 

 As falas dos participantes destacam que uma cultura de segurança fortalecida contribui para um clima favorável de trabalho, conforme ilustram os excertos: *Entendem e sabem a importância das ferramentas [checklist da cirurgia segura], só pulam etapas devido à correria, erro não é sozinho, são várias etapas não realizadas. O óbvio não é óbvio (P6, GF 1, 06/11/2020). Equipes médicas com resistência quanto aos protocolos [preenchimento dos termos de consentimento cirúrgico e anestésico] (P4, GF 1, 16/11/2020).*

 A Figura 1 mostra o *checklist* da cirurgia segura na instituição investigada, que é preenchido no sistema por etapas: antes da indução anestésica com a participação do paciente, antes da incisão cirúrgica e antes de o paciente deixar a sala operatória. 

**Figura 1:** - Foto autoral da utilização do *checklist* da cirurgia segura

 Além disso, está afixado nas salas cirúrgicas um quadro com as informações mais importantes a serem preenchidas para cada procedimento cirúrgico, que orienta a realização das etapas do *time out* (pausa cirúrgica) e *sign out* (antes de o paciente sair da sala cirúrgica), conforme ilustra a Figura 2 . 

**Figura 2:** - Foto autoral do quadro de informações do *checklist* fixado nas salas cirúrgicas

 Existem iniciativas da instituição para valorizar os princípios da segurança por meio da implementação dos quadros para o *checklist* da cirurgia segura e de murais para disseminar as informações institucionais e setoriais. Tais aspectos são abordados nos GF e na CF. 

 Os participantes destacam que muitas equipes médicas se negam a aplicar os termos de consentimento cirúrgico e anestésico, alegando falta de tempo. Este mesmo argumento é utilizado para a não realização do *checklist* da cirurgia segura, sendo que, segundo os profissionais de enfermagem mencionam, este procedimento demanda dois ou três minutos. 

A categoria 2 aborda os temas de acessibilidade dos gestores, informação compartilhada e empatia. Destaca-se que a comunicação entre as equipes médicas e de enfermagem transcorre de maneira tranquila, e que a interação cotidiana entre os trabalhadores facilita o processo de comunicação.

 Nos GF, os participantes ratificam essa fragilidade, não identificando uma liderança presente e atuante nos CC que apresentam menores escores. Por outro lado, nas discussões, profissionais dos CC com pontuações positivas destacam a acessibilidade da liderança do setor e o reconhecimento profissional como elementos importantes para o trabalho em equipe, como nas falas a seguir: *[...] alguns não têm acesso para resolver, deveria ter mais reuniões para expor o que o grupo quer falar, sentimos falta disso (P4, GF 1, 06/11/2020). Concordo com mais reuniões (P1, GF 2, 06/11/2020).*

 O quadro de pessoal insuficiente também é mencionado nas discussões em grupo, vinculado ao *turnover* elevado da instituição, por falta de reconhecimento e retenção dos profissionais. Os colaboradores manifestam que se sentem sobrecarregados e esgotados, sentimento verbalizado por eles: *Falta responsabilidade dos médicos, anestesistas, melhorar o dimensionamento da enfermagem, diminuir o absenteísmo (P6, GF 1, 06/11/2020). Resistência dos médicos nos processos (P4, GF 1, 06/11/2020). Não respeitam [médicos] (P1, GF 1, 06/11/2020). [...] estão saindo por falta de equipe, coleguismo, estamos perdendo muitas pessoas boas, falta de reconhecimento, motivação. O ruim é elogiado e o bom é desmotivado (P4, GF 2, 06/11/2020). Pessoas se sentem sobrecarregadas, as pessoas estão esgotadas, cansadas (P2, GF 2, 06/11/2020).*

Nesse trecho de relatos, além de sobrecarga e esgotamento, nota-se a percepção de pouco engajamento de outras categorias, como por exemplo a médica, e que merece destaque. Ademais, observa-se que sentimentos relacionados com a falta de reconhecimento e motivação exercem influência importante na dedicação e execução do trabalho. Estes últimos têm intrínseca ligação com o papel das lideranças de enfermagem, e, normalmente, as melhorias não geram custos, por isso seria oportuno explorar esse aspecto.

 Também relatam sentir falta de reuniões com as equipes como estratégia para compartilhar as informações entre os profissionais de saúde e contribuir para a segurança do paciente. Entre as ações implementadas e apontadas nas CF está a utilização de murais de informação, cujo objetivo é facilitar a comunicação ( Figura 3 ). Os participantes citam os murais no corredor e nos espaços de descanso como um importante recurso para a transmissão das informações institucionais, tais como resultados econômico-financeiros, indicadores de qualidade e eventos adversos mensais, conforme as manifestações: *Acho que comunica muito [mais], importante que [profissionais] saibam as metas (P1, GF 2, 11/02/2021). Eles [profissionais] gostam de saber os resultados (P2, GF 2, 11/02/2021). Precisamos nos aproximar mais dos técnicos, passando as metas, o que alcançamos no mês (P1, GF 2, 11/02/2021).*

 Os excertos das manifestações dos participantes que fundamentam as categorias temáticas estão apresentados na *joint display* ( Figura 4 ) de forma integrada com os dados quantitativos, e buscam subsidiar a compreensão dos resultados obtidos pelo questionário SAQ/CC. 

**Figura 3:** - Foto autoral do mural de informações

**Figura 4:** - Integração dos resultados quantitativos e qualitativos por meio de *joint display*

**Resultados quantitativos**	**Resultados qualitativos**	**Integração**
Domínio 1: Clima de Segurança – na análise geral obtém escore acima de 78,5, denotando uma cultura de segurança positiva. Apresenta diferença estatisticamente significativa entre os cinco CC ^†^ agrupados (p=0,047). O CC ^†^ F ^‡^ obtém pontuação negativa (71,4), e o CC ^†^ G ^§^ apresenta a pontuação limítrofe de 75,0. Os demais estão acima do ponto de corte.	Discute-se nos GF ^*^ estratégias como a reformulação do *checklist,* facilitando o preenchimento das informações e evitando desperdício de tempo com elementos irrelevantes.	Um avanço no sentido de fortalecer uma cultura de segurança foi observado nas ações descritas nos GF ^*^ e nas caminhadas fotográficas, ao destacarem melhorias das informações nos murais, assim como a implementação dos quadros com o *checklist* da cirurgia segura. Essas estratégias são vistas como um recurso para difundir a cultura de segurança do paciente na instituição e ajudam a explicar a percepção positiva obtida pelos escores na maioria dos CC ^†^ no domínio Clima de Segurança.
Domínio 2: Percepção da Gerência – na análise global é um dos domínios com menor pontuação (mediana 70) entre os CC ^†^ . Os CC ^†^ F ^‡^ , G ^‡^ e H ^‡^ apresentam fragilidades com escores abaixo de 75 pontos. Os CC ^†^ agrupados A+B ^§^ e C+D+E ^‡^ apresentam percepções positivas. Na comparação entre os grupos, verificou-se que o CC ^†^ F ^‡^ tem a menor mediana (62,5), com diferença significativa (p=0,016) em relação aos CC ^†^ A+B ^‡^ (80) e C+D+E ^‡^ (80).	Esse domínio aborda a aprovação ou desaprovação das ações das lideranças relacionadas com as questões de segurança.	A Percepção da Gerência apresenta fragilidades na maioria dos CC ^†^ em ambas as etapas. O perfil de liderança pouco presente e atuante é aspecto de destaque nos CC ^†^ com menores escores. Este domínio pode ser melhorado a partir de conversas individuais, *feedbacks* e reuniões de alinhamento com os gestores. Tal aspecto foi destacado pelos participantes do GF ^*^ (grupo B) ao mencionarem não identificar a presença do gestor no ambiente de cuidado.
Domínio 4: Condição de Trabalho – apresenta pontuação de 68,7 entre os CC ^†^ . Na análise global, é o domínio com menor pontuação, indicando maiores fragilidades na construção da cultura de segurança nos CC ^†^ avaliados. Apenas o CC ^†^ G ^§^ apresenta pontuação favorável (83,3), com diferença significativa (p=0,001) em relação aos CC ^†^ A+B ^‡^ e F ^‡^ , ambos com 66,6. Os demais CC ^†^ estão com pontuações limítrofes ou abaixo do ponto de corte 75.	Este domínio relaciona-se com a percepção da qualidade do ambiente de trabalho pelos profissionais. Nos GF ^*^ são sinalizadas a sobrecarga de trabalho, vinculada à dinâmica intensa no dia a dia do CC ^†^ . Também se destaca a importância do auxílio entre os profissionais.	A Condição de Trabalho apresenta percepção negativa entre os CC ^†^ na etapa quantitativa, dado reforçado nas manifestações dos participantes. A falta de pessoal e a dinâmica intensa de trabalho acarretam sobrecarga ao trabalhador. Existem oportunidades de melhoria através de um redimensionamento de equipes, estímulo da colaboração entre os pares e revisão das escalas de atividades diárias.
Domínio 5: Comunicação no Ambiente Cirúrgico – tem melhores pontuações no geral e em todos os CC ^†^ , e demonstra percepção positiva quanto à qualidade da comunicação e colaboração na equipe multiprofissional. Na pontuação geral, apresenta mediana de 81,2. Apenas o CC ^†^ F ^‡^ apresentou pontuação limítrofe, 75,0, sem diferença significativa entre os grupos (p=0,101).	Os participantes destacam a importância da interação entre as equipes de saúde como fundamental para o trabalho diário, juntamente com a comunicação.	Apesar da pontuação positiva na etapa quantitativa, é destaque na etapa qualitativa a necessidade de um maior número de reuniões e interação entre as equipes de saúde. Mesmo identificando-se que a utilização do *WhatsApp* colabora para a agilidade da comunicação entre as equipes, espaços de troca e diálogos podem aprimorar a comunicação e fortalecer a cultura de segurança.

† CC = Centro Cirúrgico;

‡ Letras de designação de cada um dos centros cirúrgicos pesquisados

* GF = Grupo focal;

 Os domínios 3 (Percepção do Estresse) e 6 (Percepção do Desempenho Profissional) na etapa quantitativa apresentam escores positivos. O domínio 3 tem o maior escore na avaliação global (81,2), enquanto o domínio 6 apresenta mediana de 75,0 na análise global. Os CC A+B e G apresentam melhores pontuações (81,2). Os demais têm pontuação limítrofe. Como esses temas não foram discutidos nos GF, esses domínios não foram incluídos na *joint display* para integração dos dados. 

## Discussão

 Os dados de caracterização da amostra do presente estudo são coerentes com outros achados no contexto brasileiro, indicando um predomínio de mulheres (74,7%), profissionais de enfermagem 126 (75,2%), com mediana de idade de 37 anos, atuando nos CC. Uma pesquisa realizada no Brasil em um CC de um hospital universitário, a fim de avaliar o risco de doenças patogênicas nestes trabalhadores, mostrou que a maior parte deste setor são mulheres (81,9%) com idades entre 36 e 50 anos, corroborando os achados deste estudo ^(^ ^21^ ^)^ . 

 A primeira parte do instrumento SAQ/CC demonstrou que apenas a categoria de enfermagem (enfermeiro, instrumentador e circulante cirúrgico) atingiu o escore mínimo (≥75), para ser considerada uma percepção positiva de cultura de segurança. Em outro estudo ^(^ ^22^ ^)^ , os enfermeiros também obtiveram pontuações médias mais elevadas em itens relacionados com a qualidade da comunicação e colaboração, de modo semelhante aos resultados deste estudo. Um estudo realizado no CC de um hospital público universitário no estado do Paraná, que encontrou uma pontuação mais baixa entre instrumentadores/circulantes, sugere que há uma baixa qualidade de comunicação e colaboração nas outras categorias que trabalham no CC ^(^ ^21^ ^)^ . 

 A comunicação e a colaboração adequadas entre os membros da equipe do CC são fundamentais para garantir a segurança do paciente durante a cirurgia, de modo que todos têm um papel importante a desempenhar, e é essencial que cada um compreenda cada função e trabalhe em conjunto ^(^ ^22^ ^)^ . 

 A comunicação eficaz entre os profissionais de saúde, pacientes e familiares é um fator crucial para a prestação de cuidados com qualidade e segurança. É a base para a tomada de decisão compartilhada, a identificação precoce de problemas e a resolução de conflitos, todos elementos fundamentais para o cuidado centrado no paciente. Nesta perspectiva, o enfermeiro assume um papel de destaque na integração das informações entre as equipes, diminuindo as chances de ocorrência de eventos adversos e promovendo uma comunicação aberta e honesta entre os membros das equipes ^(^ ^23^ ^-^ ^24^ ^)^ . 

 Quando a cultura de segurança é estabelecida em uma instituição, a comunicação entre os profissionais se torna mais fácil e eficaz. Isso ocorre porque há uma maior confiança entre os membros da equipe, que se sentem mais à vontade para se comunicar abertamente e compartilhar informações relevantes. Além disso, quando todos os membros da equipe estão cientes das medidas de segurança adotadas e compreendem a importância de segui-las, o trabalho em equipe e a colaboração são aprimorados, o que contribui para a prestação de um cuidado de qualidade ao paciente ^(^ ^25^ ^)^ . 

A análise descritiva geral do escore por agrupamento de CC, a partir dos domínios do SAQ/CC, revelou uma percepção favorável do clima de segurança nos locais considerados. Isso indica uma percepção positiva com relação à segurança do paciente nesses ambientes. Entre os domínios do SAQ/CC, Percepção da Gerência e Condição de Trabalho se destacaram como os mais frágeis nos CC avaliados. Isso sugere que os colaboradores desses centros podem ter uma percepção menos positiva sobre gestão e as condições de trabalho existentes. É possível que haja uma necessidade de melhorias nesses aspectos para promover uma cultura de segurança mais sólida.

Por outro lado, os domínios Comunicação no Ambiente Cirúrgico, Clima de Segurança, Percepção do Desempenho Profissional e Percepção do Estresse apresentaram pontuações favoráveis. Isso indica que os colaboradores dos CC têm uma percepção positiva nesses quesitos, o que sugere a existência de uma cultura de segurança mais robusta e uma boa comunicação entre os profissionais envolvidos.

 A análise integrada dos resultados reforça a fragilidade dos domínios Percepção da Gerência e Condição de Trabalho nos CC avaliados, tanto pelas pontuações negativas quanto pelas manifestações dos profissionais nos grupos focais. É interessante observar que em outros estudos também foram encontrados resultados semelhantes com relação às percepções gerenciais e condições de trabalho nos CC. Essa consistência nos resultados sugere que a baixa percepção sobre a gerência, juntamente com as condições de trabalho desfavoráveis, pode levar os profissionais de saúde a desconhecerem o compromisso da gestão das instituições com os fatores ocultos da cultura de segurança. Consequentemente, essa falta de conhecimento pode se refletir em escores negativos e indicar um distanciamento entre a equipe e seus superiores ^(^ ^26^ ^-^ ^27^ ^)^ . 

 Para proporcionar o desenvolvimento de uma cultura de segurança do paciente, é necessário investir e desenvolver treinamentos e engajar os profissionais de saúde com esta temática. Além disso, é fundamental que os hospitais forneçam os recursos de apoio necessários para os profissionais de saúde ^(^ ^28^ ^)^ . 

A pontuação positiva no domínio Clima de Segurança foi reforçada pelas falas dos participantes na etapa qualitativa, destacando a importância de uma cultura de segurança fortificada para criar um clima de trabalho favorável.

 É interessante notar que um estudo realizado em três hospitais públicos e privados no estado do Rio Grande do Sul comparou o clima de segurança dessas instituições, e identificou pontuações positivas no serviço privado, enquanto as instituições filantrópicas apresentaram escores abaixo da média, evidenciando uma fragilidade na cultura de segurança nessas instituições ^(^ ^29^ ^)^ . 

Essas descobertas destacam a importância de uma cultura de segurança sólida para promover um clima de trabalho positivo e seguro nos CC. Uma cultura de segurança estabelecida é essencial para encorajar a comunicação aberta, a aprendizagem contínua e o engajamento de todos os profissionais de saúde na identificação e prevenção de eventos adversos.

 O domínio Percepção do Estresse também apresentou uma pontuação acima da média em outro estudo, indicando que os participantes percebem fatores estressores no ambiente de trabalho ^(^ ^30^ ^)^ . Essa percepção pode ser especialmente relevante para os profissionais que atuam em CC, uma vez que estão expostos a uma série de estresses devido ao ambiente de trabalho desafiador. 

 Os profissionais que trabalham em CC enfrentam níveis elevados de estresse devido a vários fatores, como ambiente fechado, riscos envolvidos, diversas rotinas, necessidade de habilidades técnicas e alta produtividade exigida ^(^ ^31^ ^)^ . Esses elementos podem contribuir para a pressão emocional e psicológica enfrentada pelos profissionais da área cirúrgica. 

 Diante desse cenário, sugere-se a reorganização das atividades e o redimensionamento das equipes como medidas para melhorar a qualidade de vida no trabalho e reforçar a segurança do paciente no ambiente cirúrgico ^(^ ^26^ ^)^ . Ao reavaliar a distribuição de tarefas e a carga de trabalho, é possível promover um equilíbrio mais saudável para os profissionais, reduzindo o estresse e aumentando o bem-estar no trabalho. 

A pontuação limítrofe no domínio Percepção do Desempenho Profissional indica uma avaliação neutra por parte da equipe cirúrgica nesse aspecto, de acordo com os resultados do SAQ/CC. Esse domínio está relacionado com a forma como o cansaço e a sobrecarga de trabalho afetam o desempenho profissional dos indivíduos.

 O desempenho profissional está intrinsecamente ligado à satisfação no trabalho, refletindo a experiência individual de cada profissional. A satisfação no trabalho é definida como um sentimento positivo com relação ao trabalho, que engloba aspectos como o conteúdo do trabalho, as oportunidades de desenvolvimento, o reconhecimento, as condições de trabalho e os relacionamentos com colegas e superiores ^(^ ^30^ ^)^ . 

É importante considerar que a satisfação no trabalho e o desempenho profissional são afetados por uma variedade de fatores, incluindo as condições de trabalho, o suporte organizacional, o reconhecimento e as oportunidades de crescimento. Portanto, é fundamental que as instituições de saúde estejam atentas a esses aspectos e adotem medidas para promover um ambiente de trabalho saudável e equilibrado, que valorize o bem-estar dos profissionais.

 A satisfação no trabalho não apenas afeta o bem-estar e a saúde dos profissionais, mas também está relacionada com a prevenção de doenças ocupacionais e o alcance de melhores resultados no processo de trabalho, que inclui as atividades desempenhadas pelos enfermeiros e demais membros da equipe de saúde ^(^ ^31^ ^)^ . 

Quando os profissionais estão satisfeitos no trabalho, eles tendem a ser mais engajados, motivados e dedicados às suas responsabilidades. Isso pode levar a uma maior eficiência, produtividade e qualidade na assistência ao paciente. Por outro lado, a insatisfação no trabalho pode levar a um ambiente negativo, desmotivação, aumento do estresse e possíveis erros ou falhas na prestação de cuidados.

Como limitações do estudo, ressalta-se o número reduzido de respondentes em algumas categorias profissionais, devido ao período crítico de saúde ocasionado pela pandemia de Covid-19 e a redução expressiva das agendas cirúrgicas. A ausência de representantes nos GF de todas as categorias investigadas na primeira etapa, entre eles médicos, perfusionistas e auxiliares de farmácia ou higienização, é uma limitação. No entanto, o estudo oferece reflexões relevantes sobre o clima e as atitudes de segurança no CC, e pode ser considerado para dar direcionamentos assertivos ao cuidado seguro.

Os resultados do estudo indicam uma melhor percepção do clima de segurança pela equipe de enfermagem, o que sinaliza um potencial maior de seu engajamento na construção de uma cultura de segurança. Neste sentido, destaca-se a necessidade de uma avaliação mais aprofundada de estratégias para o engajamento das equipes médicas, com o objetivo de promover um trabalho interprofissional e colaborativo.

As contribuições deste estudo para a prática assistencial incluem o destaque à importância de desenvolver os profissionais que ocupam cargos de gestão, buscando uma maior aproximação e compreensão das necessidades da equipe assistencial. Isso implica em um olhar mais assertivo e sensível para promover um ambiente de trabalho saudável e seguro.

Além disso, o estudo destaca a necessidade de investir nas condições de trabalho oferecidas aos colaboradores. Isso envolve a adequação da sobrecarga laboral, considerando uma distribuição equitativa das tarefas, e a revisão do dimensionamento de pessoal. Ao fornecer uma carga de trabalho adequada, os centros cirúrgicos podem promover um clima de segurança mais positivo, reduzindo o risco de erros e melhorando a qualidade do cuidado prestado. Essas sugestões têm como objetivo melhorar a qualidade da assistência oferecida, promovendo um ambiente de trabalho mais colaborativo, seguro e eficaz para toda a equipe assistencial.

## Conclusão

Os CC estudados apresentaram resultados que indicam uma percepção positiva do clima de segurança entre os profissionais de saúde e de apoio. Os domínios Percepção do Estresse, Comunicação no Ambiente Cirúrgico, Clima de Segurança e Percepção do Desempenho Profissional obtiveram pontuações mais elevadas em relação à cultura de segurança, dado que foi reforçado na análise qualitativa.

A análise feita nesta pesquisa sobre as atitudes de segurança nos CC proporcionou uma compreensão aprofundada dos desafios enfrentados nesse ambiente complexo. A combinação de dados quantitativos e qualitativos, que é uma abordagem ainda pouco explorada na enfermagem perioperatória, mostrou-se desafiadora, porém profícua.

Ainda que a análise global do domínio Comunicação e Colaboração entre as Equipes tenha se mostrado positiva, tanto nos dados quantitativos quanto nos qualitativos foram identificadas oportunidades de melhorias nesse processo. A equipe de enfermagem parece aderir mais facilmente aos processos e rotinas institucionais voltados à segurança do paciente, o que reflete maior fortalecimento cultural desta categoria no que diz respeito ao cuidado seguro.
